# State-dependent binding of the wedge domain controls inactivation of the mechanosensitive ion channel PIEZO1

**DOI:** 10.1038/s41467-026-76927-0

**Published:** 2026-08-26

**Authors:** Lucas Roettger, Nadja Zeitzschel, Christian Gorzelanny, Clement Verkest, Stefan G. Lechner

**Affiliations:** 1 Department of Anesthesiology, University Medical Center Hamburg-Eppendorf, Hamburg, Germany; 2 Department of Dermatology and Venereology, University Medical Center Hamburg-Eppendorf, Hamburg, Germany

**Keywords:** Ion channels in the nervous system, Neurophysiology, Ion channels, Permeation and transport

## Abstract

The mechanically activated ion channel PIEZO1 transduces membrane tension into intracellular calcium signals and is critical for a wide range of physiological processes. Recent structural and functional studies have established a detailed framework for PIEZO1 activation, but the molecular mechanisms governing its rapid inactivation remain incompletely understood. Here, we examine the contribution of the intracellular wedge domain to PIEZO1 inactivation using site-directed mutagenesis, electrophysiological recordings and MINFLUX nanoscopy. We show that wedge deletion and disruption of specific π-π and cation-π interactions between the wedge α1-helix and the pore module diminishes inactivation without impairing channel activation. Moreover, MINFLUX nanoscopy suggests that the wedge stabilizes a flat inactivated conformation of PIEZO1 and suggests that wedge dissociation is required for recovery from inactivation. Together, our data support a mechanism with the wedge acting as a state-dependent inactivation particle that docks to the pore module to terminate channel activity during sustained mechanical stimulation.

## Abstract (web summary)

Inactivation of PIEZO1 in the presence of mechanical stimulation is essential for normal physiological function. Here, Roettger et al. show that the wedge domain binds to the flat conformation of PIEZO1 thereby stabilizing the flat state and controlling recovery from inactivation.

## Introduction

PIEZO1 is a mechanically activated ion channel that converts membrane tension into rapid calcium influx, enabling cells to sense and respond to forces in their environment, such as shear stress, tissue stretch, and cell compression^1,2^. Accordingly, PIEZO1 is essential for diverse physiological processes, including vascular development, blood pressure regulation, erythrocyte volume control, chondrocyte mechanotransduction, and cellular migration^3–5^. Cryogenic electron microscopy (cryo-EM) structures revealed that PIEZO1 assembles as a homotrimer with a distinctive triskelion architecture, comprising three curved peripheral “blades” that emerge from a central pore module, which consists of an inner (IH) and an outer helix (OH) as well as a large extracellular domain known as the cap^6–8^. The ion conduction pathway of PIEZO1 contains several gates including (from top to bottom) a cap gate, a transmembrane gate, and possibly a lateral plug gate^9–11^. Accordingly, the choreography of gating motions and allosteric rearrangements that eventually lead to the opening of the ion-conduction pathway are complex and involves several critical steps. Cryo-EM structures obtained from different conformational PIEZO1 states^12,13^ together with in-silico modelling^14,15^, high-speed atomic force microscopy (AFM)^16^ and MINFLUX nanoscopy of PIEZO1 in its native environment^17–19^ have established a mechanistic framework that suggests that PIEZO1 adopts a curved conformation in the closed state, in which the blades are tilted upwards, and transitions into a flat conformation upon mechanical and chemical activation. The blades are connected to the pore module by the so-called beam – a long intracellular α-helix – which appears to act as a lever that relays the flattening motions of the blades to the pore module to promote channel activation, possibly by opening the lateral plug gate^11,20,21^. The triangular-shaped anchor domain, which resides within the plasma membrane and between the pore module and the blade, however, also appears to be involved in coupling blade flattening to the pore, as subtle changes in its geometry have profound effects on PIEZO1 sensitivity and inactivation^22^. Finally, the extracellular cap domain appears to play a central role in PIEZO1 activation. Upon flattening, the cap moves downwards, thereby compressing the IH- and OH-to-cap linkers, which opens the lateral cap gates such that ions can enter the central ion conduction pathway^9,13,23^.

A key property of PIEZO1 is its rapid inactivation in the presence of sustained mechanical stimulation, which appears to be essential for normal physiological function, as mutations that slow down inactivation have been linked to human diseases such as hereditary stomatocytosis and xerocytosis^3,4,24–28^. Moreover, modulation of inactivation appears to be an effective strategy to fine-tune and adjust PIEZO1-function in a cell type-specific manner by increasing cumulative calcium entry and thereby potentiating downstream signalling cascades. Thus, numerous PIEZO1 protein interaction partners, including TMEM150c^29^, CADM1^30^, MDFIC and MDFI^31^, MDFIC2^32,33^, as well as lipids, such as ceramide^34^, poly-unsaturated fatty acids^35^, and possibly phosphatidylinositol 4,5-bisphosphate^36^ were shown to slow inactivation. The choreography of the allosteric rearrangements that cause inactivation and hence the mechanism by which it is altered by the afore-mentioned interactions are, however, only poorly understood. Seminal work by the Grandl lab^37–39^ together with a recent study from Liu and colleagues^9^ suggests that the cap domain plays a central role in inactivation. Thus, replacing specific subdomains in the cap of PIEZO1 with the equivalent domains of PIEZO2 confers rapid PIEZO2-like inactivation to PIEZO1^37,38^. Moreover, Liu et al. proposed that the flexible linkers that connect the cap to the inner and outer helix of the pore domain may serve as compressive springs that store energy upon channel flattening and subsequently release it to inactivate the channel by closing the cap gate^9^. There is, however, compelling evidence suggesting that inactivation is more complex and involves additional allosteric mechanisms. Lewis et al., for example, described two kinetically distinct inactivated states^39^, Zheng et al. found a hydrophobic inactivation gate in the inner pore^10^, and Wu and colleagues discovered a positively charged residue in the inner helix that controls voltage-sensitivity of inactivation^38^. Moreover, disease-causing mutations that slow inactivation are not restricted to the cap and the IH but were also found throughout various other domains of PIEZO1, including the blades, the beam and the anchor^26^.

**Fig. 1:**
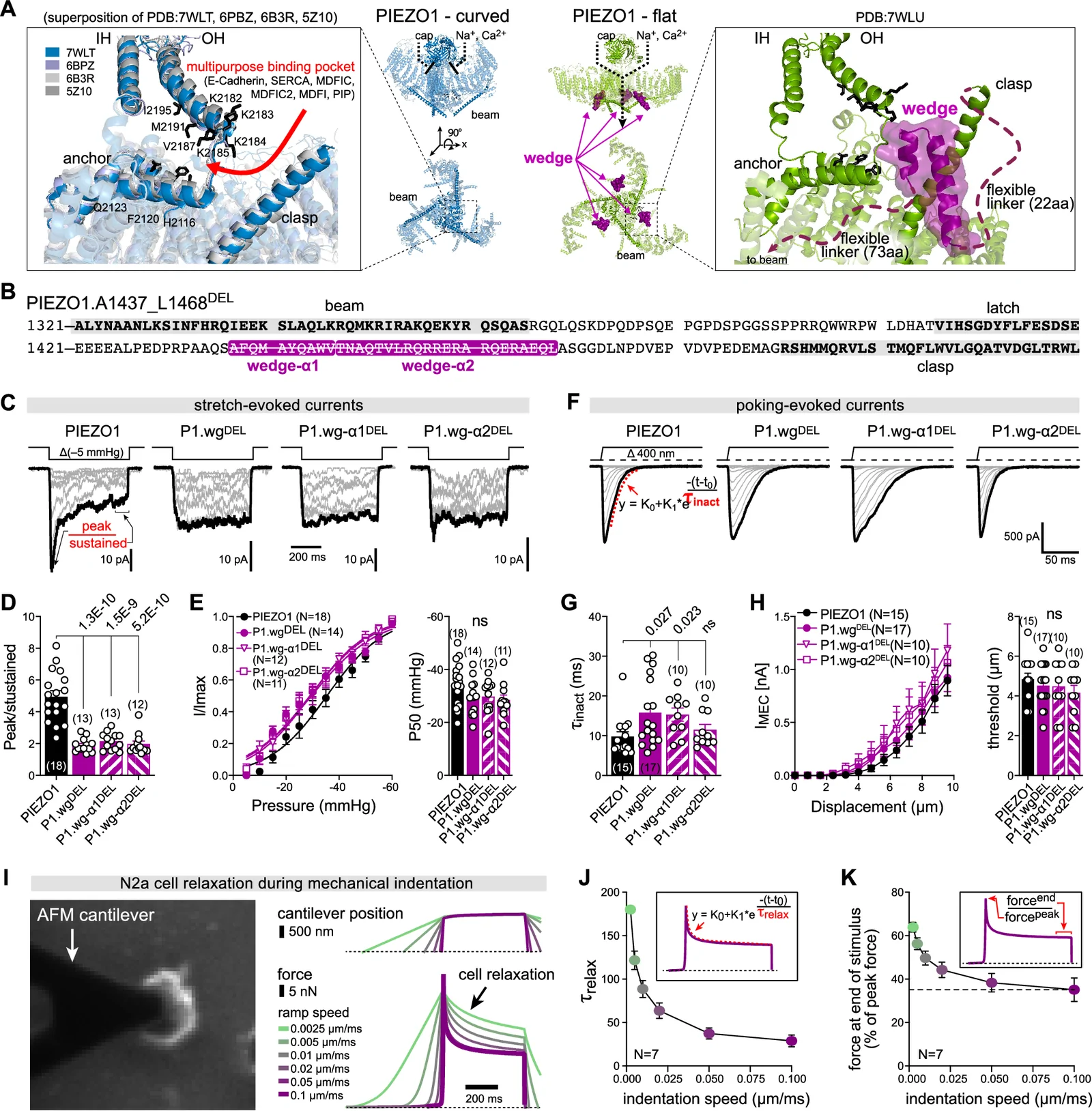
Wedge deletion abolishes inactivation of pressure-evoked PIEZO1 currents. **A** Superposition of the indicated curved (left) and flat (right) cryo-EM structures of PIEZO1. Note, the wedge (purple) is absent from all curved structures but was resolved in close proximity to a putative multipurpose binding site in the cavity between the anchor and the outer helix (OH). **B** Partial amino acid sequence of PIEZO1 highlighting key domains (beam, latch, wedge and clasp). **C** Representative examples of stretch-evoked currents recorded from N2a-P1KO cells expressing PIEZO1 or full and partial wedge deletion mutants. **D** Comparision of peak/sustained ratios of the indicated stretch-evoked currents using the Kruskal-Wallis test. **E** Normalised pressure-response curves of the indicated channels (left) and comparison of P50 values (right) using one-way ANOVA with Dunnett’s multiple comparisons test. **F** Representative examples of poking-evoked currents recorded from N2a-P1KO cells expressing PIEZO1 or full and partial wedge deletion mutants. **G** Comparison of inactivation time constants of poking-evoked currents, determined by exponential decay fits (see F), using the Kruskal-Wallis-test with Dunn’s multiple comparison test. (**H**) Displacment–response curves (left) and comparison of mechanical activation thresholds of poking-evoked currents mediated by the indicated channels, using one-way ANOVA with Dunnett’s multiple comparison test. **I** Representative brightfield image of the AFM cantilever over a N2a-P1KO cell (left) and corresponding force measurement (right, bottom) upon cantilever displacement at the indicated velocity (top). **J** Average cell relaxation time constant (exponential fit equation in inset) at different indentation speeds. **K** Average force measured at the end of the indentation, relative to the peak at the beginning, and at different indentations. All bars in D-H represent means ± s.e.m., with white circles showing individual values of biological replicates and *P*-values being provided above bars. Numbers of biological replicates (individual cells examined) are indicated in brackets in panels (**D**–**H**,** J** and **K**).

Here, we examine the role of the wedge domain in PIEZO1 inactivation. The wedge domain is a short intracellular domain (32 amino acids) comprising two α-helices (α1 and α2) that are linked to the C-terminus of the beam and the N-terminus of the clasp via long flexible linkers (73 and 22 amino acids; Fig. 1A, Supplementary Fig. 1A, B). An intriguing observation regarding the wedge, which sparked our interest in this domain, was that the structure of the wedge had not been resolved by any published cryo-EM structure of PIEZO1 in the curved conformation^6–8^ and the intermediate flat conformation, potentially representing an open state^12^ (Fig. 1A and Supplementary Fig. 2A), suggesting high mobility of the wedge in the closed state. In the flat inactive state, however, the wedge was resolved and appears to dock between the clasp, outer helix, and anchor domains^13^ – a region enriched for binding sites for regulatory proteins and lipids (Fig. 1A and Supplementary Fig. 1C). Thus, interaction partners such as E-cadherin^40^, SERCA^41^, TMEM150C^29^, MDFIC and MDFI^31^, MDFIC2^32,33^ and phosphoinositides^36^ bind within or adjacent to this region, highlighting it as a structural hub for tuning PIEZO1 function. In this study, we investigate the role of the wedge domain in PIEZO1 function using site-directed mutagenesis and electrophysiology, complemented by MINFLUX super-resolution microscopy.

## Results

### The wedge domain controls inactivation of PIEZO1

To examine the role of the wedge domain in PIEZO1 function, we generated mutant channels that lack the full wedge (PIEZO1.A1437_L1468^DEL^), hereafter referred to as P1.wg^DEL^, as well as partial wedge deletions that lack either the α1- or the α2-helix of the wedge (P1.wg-α1^DEL^ and P1.wg-α2^DEL^; Fig. 1B) and characterised mechanically-evoked currents as well as chemically-induced calcium transients mediated by these channels using patch-clamp electrophysiology and GCamp8 calcium imaging, respectively. The mutant channels were expressed in Neuro2a-PIEZO1 knockout cells (N2a-P1KO)^42^ and mechanotransduction currents were evoked by pressure-induced membrane stretch in the cell-attached patch-clamp configuration (i.e., pressure-clamp technique) or by focal mechanical stimulation with a small glass rod in the whole-cell patch-clamp mode (poking technique^43^, see reference ^44^ for comparison of the two techniques). All three mutant channels were trafficked to the plasma membrane (Supplementary Fig. 2B) and produced robust mechanically activated currents in the pressure-clamp and poking assay. Notably, while the three mutants were indistinguishable from full-length PIEZO1 with regards to current amplitudes, activation thresholds and single channel conductance, they exhibited significantly slower inactivation during sustained mechanical stimulation (Fig. 1C–H and Supplementary Fig. 2C–E). Thus, the ratios of peak current amplitudes to sustained current amplitudes in pressure-clamp recordings (see Fig. 1C, D) significantly decreased from 4.95 ± 0.37 (PIEZO1; mean ± s.e.m; *N* = 18) to 1.89 ± 0.12 (P1.wg^DEL^; *N* = 13, *P* = 3.67E-6; Kruskal-Wallis test), 2.15 ± 0.15 (P1.wg-α1^DEL^; *N* = 13, *P* = 2.15E-4) and 1.97 ± 0.19 (P1.wg-α2^DEL^; *N* = 13, *P* = 9.63E-6) upon full or partial deletion of the wedge domain. Likewise, the inactivation time constants of poking-evoked currents, determined by exponential decay fits (Fig. 1F, G), increased from 9.8 ± 1.2 ms (PIEZO1, *N* = 15) to 15.8 ± 2.1 ms (P1.wg^DEL^; *N* = 17, *P* = 0.027; Kruskal-Wallis test) and 15.3 ± 1.6 (P1.wg-α1^DEL^; *N* = 10, *P* = 0.024; Kruskal-Wallis test) in the absence of the full wedge and the wedge-α1 helix but not upon deletion of the wedge-α2 helix (11.5 ± 1.4 ms; *N* = 10, *P* = 0.83; Kruskal-Wallis test).

Interestingly, wedge deletion affected PIEZO1 inactivation less strongly in the poking assay than in the pressure-clamp assay. Thus, albeit significantly slower, poke-evoked P1.wg^DEL^ and P1.wg-α1^DEL^ currents still showed substantial decay during sustained mechanical stimulation, whereas stretch-evoked currents from the same mutants did not (compare Fig. 1D, G). However, in the poking assay, current decay cannot be unambiguously attributed to inactivation, defined as the transition from the active to an inactive state during continued stimulation. It may also reflect deactivation, i.e., transition from the active to the closed/resting state, because the effective stimulus declines over time due to relaxation of the indented cell, such that the forces may drop below the activation threshold. This limitation had previously been proposed by Lewis and Grandl in a comprehensive comparison of the stretch- and poke assays^44^ and was subsequently demonstrated by Young et al., who observed a rapid decay of indentation forces during mechanical indentation of HEK293 cells using atomic force microscopy (AFM)^45^.

To determine whether force relaxation could contribute to the residual decay of poke-evoked P1.wg^DEL^ and P1.wg-α1^DEL^ currents, we measured relaxation of N2a-P1KO cells during ramp-and-hold indentation using AFM. Cells were indented with tipless AFM cantilevers using ramp speeds from 0.0025 to 0.1 µm/ms, while indentation forces were monitored throughout the stimulus (Fig. 1I). These experiments showed that indentation forces indeed rapidly decline during the static phase of stimulation in a ramp-speed-dependent manner, levelling off at 35.2 ± 14.5 % of the initial peak force with a time constant of 28.9 ± 18.5 ms at the fastest tested ramp speed (Fig. 1I–K). Higher ramp speeds could not be analysed because they produced hydrodynamic drag forces during the approach phase that confounded force measurements. Nevertheless, because both the rate and magnitude of force relaxation increased with ramp speed, indentation force decay is likely to be even more pronounced at the 1 µm/ms indentation speed commonly used in the poking assay.

Together, these observations suggest that during poke stimulation, indentation forces may rapidly decline below the activation threshold, especially in membrane regions distant from the site of indentation that experience smaller peak forces in the first place. Thus, deactivation may contribute to the residual current decay observed for poke-evoked P1.wg^DEL^ and P1.wg-α1^DEL^ currents. Given this limitation of the poking assay, we used the pressure-clamp assay for most subsequent experiments assessing PIEZO1 inactivation.

Consistent with the slowed inactivation, which prolongs the time the channels spend in the open state and thus allows more calcium to enter the cell, calcium transients triggered by the selective PIEZO1 activator Yoda-2 were also larger and detectable at lower Yoda-2 concentrations in N2a cells transfected the wedge-mutants as compared to full-length PIEZO1 (Supplementary Fig. 2F). Similar to poking-evoked currents, deletion of the wedge-α1 helix had a more pronounced effect on calcium transients than wedge-α2 deletion.

Together, these data suggest that the wedge domain plays a crucial role in controlling the inactivation of PIEZO1 and suggest that this modulatory effect is predominantly mediated by the wedge α1-helix.

### The wedge binds to the anchor-OH-linker via hydrophobic interactions

**Fig. 2:**
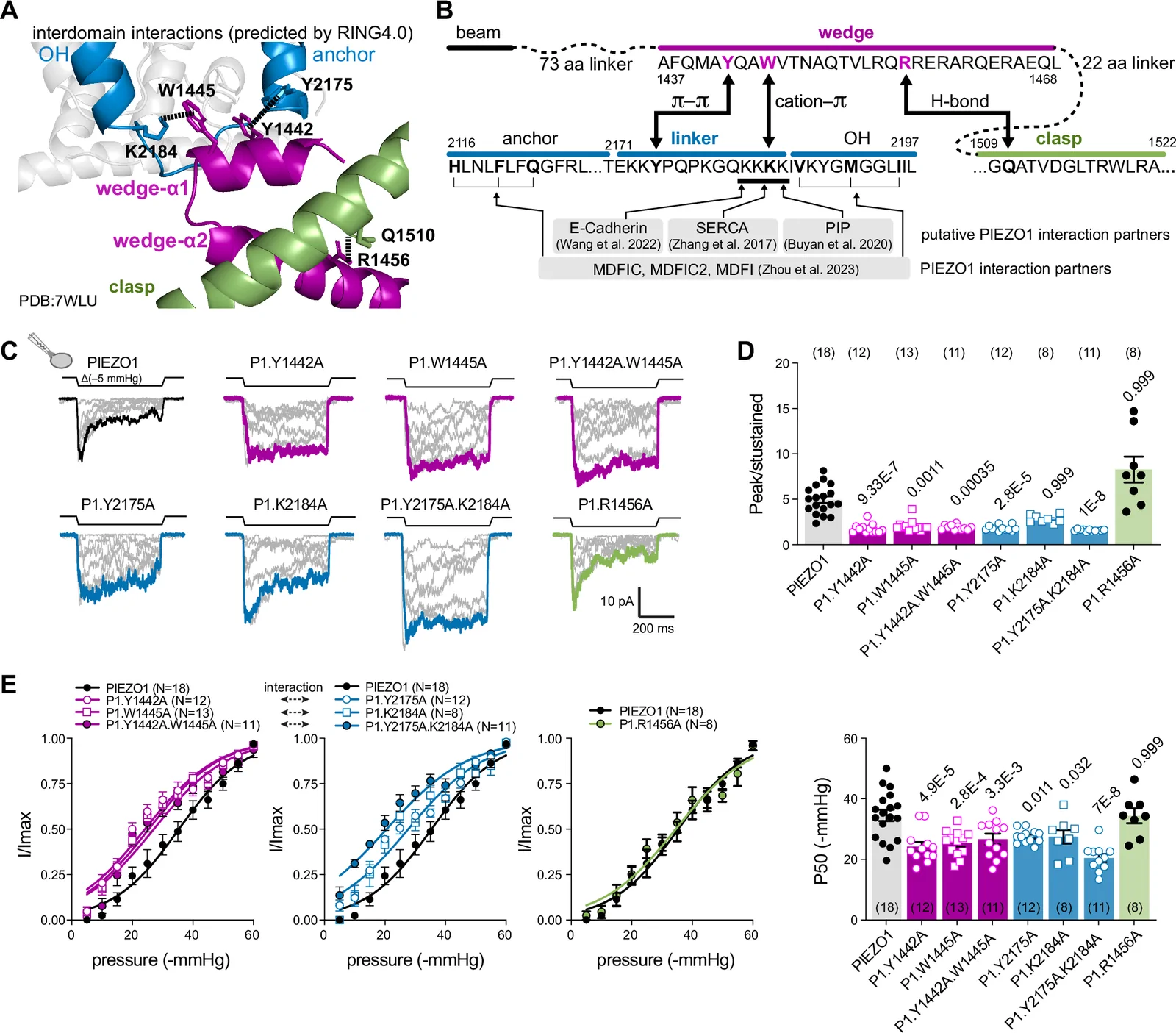
Non-covalent interactions between the wedge α1 helix and the anchor-OH linker are required for wedge docking. **A** Close-up of the anchor-OH linker region in the flat PIEZO1 structure (PDB: 7WLU) with putative interactions with the wedge as predicted by RING4.0. **B** Partial amino acid sequence of PIEZO1 showing the wedge, anchor, linker, OH and clasp domains and amino acids engaged in the putative interdomain interactions and interactions with other proteins highlighted in bold letters. **C** Representative examples of stretch-evoked currents recorded from N2a-P1KO cells expressing PIEZO1 or the indicated point mutants. **D** Comparison of peak/sustained ratios of the indicated stretch-evoked currents using Kruskal-Wallis with Dunn’s multiple comparison test. **E** Normalised pressure-response curves of the indicated channels fitted with Boltzmann sigmoidal functions (left) and comparison of P50 values (pressure required for half-maximal channel activation, right) using one-way ANOVA with Dunnett’s multiple comparisons test. All bars in (**D**, **E**) represent means ± s.e.m., with white circles showing individual values of biological replicates and *P*-values being provided above bars. The number of biological replicates (individual cells examined) are indicated in brackets in panels (**D**, **E**).

To test this hypothesis, we next analysed possible non-covalent interactions between the wedge and adjacent domains in the flattened PIEZO1 structure (PDB:7WLU)^13^ using the RING4.0 platform^46^. This analysis revealed that tyrosine 1442 (Y1442) and tryptophane 1445 (W1445) of the wedge α1 helix are in close proximity to Y2175 and K2184 of the anchor-OH-linker and thus possibly dock the wedge to the pore module via cation-π and π–π interactions, respectively (Fig. 2A). Moreover, this analysis suggested that R1456 of the wedge α2-helix forms a hydrogen bond with Q1510 of the nearby clasp domain to further stabilise wedge binding. To examine the possible role of these non-covalent interactions in PIEZO1 inactivation, we generated PIEZO1 single and double mutants in which the afore-mentioned amino acids were substituted by alanines. Strikingly, all mutants that altered possible interactions between the wedge α1-helix and the anchor-OH-linker, significantly (except K2184A) slowed down inactivation (peak/sustained ratio, PIEZO1 = 4.95 ± 0.37, *N* = 18; P1.Y1442A = 1,79 ± 0.14, *N* = 12; P1.W1445A = 2.09 ± 0.17, *N *= 1 3; P1.Y1442A.W1445A = 1.91 ± 0.07, *N *= 11; P1.Y2175A = 1.84 ± 0.08, *N *= 12; P1.K2184A = 2.86 ± 0.15, *N* = 8; P1.Y2171A.K2184A = 1.59 ± 0.03, *N* = 11; P1.R1456A = 8.29 ± 0.14, *N* = 8), whereas disrupting the possible wedge-clasp interaction by mutating R1456 did not have any effect (Fig. 2C, D). Interestingly, unlike full and partial deletion of the wedge, which did not alter significantly PIEZO1 sensitivity, abrogation of possible wedge–anchor-OH-linker interactions also shifted the pressure-response curves towards less negative pressures (Fig. 2E) and thus significantly reduced the mean pressures required for the half-maximal activation of the channels (P_50_) from 34.6 ± 1.9 mmHg in PIEZO1 (*N* = 18) to values around ~ 25 mmHg (P1.Y1442A = 24.2 ± 1.6 mmHg, *N* = 12; P1.W1445A = 25.4 ± 1.2 mmHg, *N* = 13; P1.Y1442A.W1445A = 26.7 ± 1.8 mmHg, *N* = 11; P1.Y2175A = 27.6 ± 0.7 mmHg, *N* = 12; P1.K2184A = 27.5 ± 2.3 mmHg, *N* = 8; P1.Y2171A.K2184A = 20.5 ± 1.3 mmHg, *N* = 11; P1.R1456A = 34.5 ± 2.4,* N* = 8; Fig. 2E). Those observations, i.e slowing of inactivation and decreased mechanical threshold for activation for the wedge α1-helix and anchor-OH-linker mutants but not for the α2-clasp mutant, were also observed with the poking assay (Supplementary Fig. 3A–D).

In summary, our patch-clamp recordings from full and partial wedge deletions as well as PIEZO1 point mutants together with previous cryo-EM data suggest that – in the flat (open) state – the wedge binds to the anchor-OH-linker via cation-π and π–π interactions, thereby promoting rapid inactivation of PIEZO1.

### PIEZO1 instantly recovers from inactivation in the absence of the wedge

**Fig. 3:**
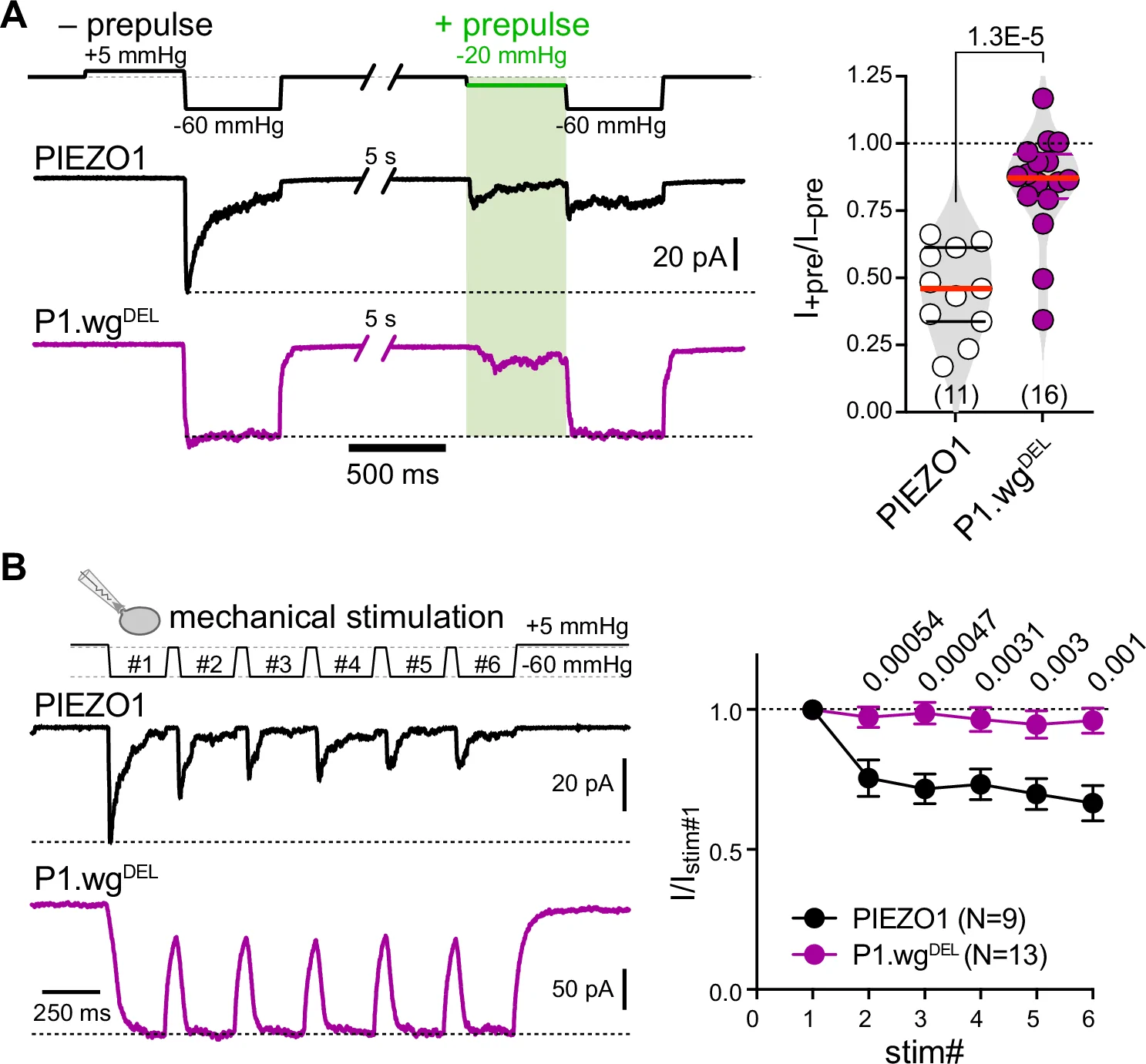
PIEZO1 instantly recovers from inactivation in the absence of the wedge. **A** Representative example traces of PIEZO1 (top) and P1-wg^DEL^ (bottom) currents evoked with (right) or without (left) a conditioning pre-pulse to − 20 mmHg and comparison of the ratios of peak current amplitudes elicited with and without conditioning pre-pulse. Circles show ratios of biological replicates, medians are indicated by horizontal red lines and were compared using two-sided Student’s upaired *t* test (*P* = 1.36E-5). **B** Example traces of PIEZO1 (top left) and P1.wg^DEL^-mediated (bottom, left) currents elicited by repetitive mechanical stimulation. Magnitude of responses normalised to the first response of PIEZO1 (black) and P1.wg^DEL^ (purple). Symbols represent means ± s.e.m. and *p*-values of multiple two-sided unpaired comparison t-tests are provided above. Numbers in brackets in panels (**A**, **B**) represent the number of biological replicates (individual cells examined).

To test if the lack of current decay during sustained membrane stretch stimulation of the P1.wg^DEL^ mutant, indeed resulted from a lack of inactivation (i.e., transition from the open to the inactivated state) rather than altered deactivation (open to closed) or adaptation (e.g., patch deterioration, seal creep^47^, cytoskeletal rearrangements^48^), we next applied two identical and supramaximal test pulses (− 60 mmHg) at an interval of 5 seconds with the second test pulse being preceded by a 500 ms conditioning pre-pulse to − 20 mmHg, which partially activates PIEZO1 and allows currents to decay back to baseline levels (Fig. 3A). In cells expressing full-length PIEZO1 the second test pulse ( + pre-pulse) consistently elicited smaller currents (I_+prepulse_/I_–prepulse_ = 0.45 ± 0.05, *N* = 11) demonstrating that a considerable fraction of PIEZO1 channels indeed transition into an inactivated state during sustained mechanical stimulation in which they are reluctant to open in response to subsequent mechanical stimuli. By contrast, P1-wg^DEL^ currents reached almost the same peak amplitudes after application of the conditioning pre-pulse currents (I_+prepulse_/I_–prepulse_ = 0.85 ± 0.05, *N* = 16), suggesting that channels do not inactivate in the absence of the wedge and immediately return to a closed and “willing” state upon stimulus removal.

We next considered recovery from inactivation. Previous studies have shown that PIEZO1 remains in an inactivated state after removal of the mechanical stimulus and that full recovery from inactivation can take up to ten seconds^39^. To test if wedge deletion alters recovery from inactivation, we applied six consecutive and supramaximal pressure pulses (magnitude = − 60 mmHg; duration = 250 ms) with varying time intervals (50 ms, Fig. 3A, 100 ms, 200 ms, 500 ms and 1000 ms; Supplementary Fig. 4A) in cell-attached recordings and measured the decay of peak current amplitudes. Consistent with previous reports^39^, full-length PIEZO1 peak current amplitudes got smaller with each stimulus – even at the longest test interval of 1000 ms – demonstrating that the channels only slowly recover from inactivation (Fig. 3B and Supplementary Fig. 4A). By contrast, the peak amplitudes of P1.wg^DEL^ currents did not decay at all and were identical throughout the entire stimulation protocol even when the consecutive test pulses were applied at extremely short intervals of 50 ms (Fig. 3B and Supplementary Fig. 4A).

### PIEZO1 remains in a flat conformation after inactivation

It is well-established that PIEZO1 adopts a curved conformation in the closed state and transitions into a flat conformation upon mechanical and chemical activation^14,16,17,19^. The allosteric mechanisms and conformational changes underlying inactivation are, however, only poorly understood. Previous studies from others and us that examined the distribution of conformational states of PIEZO1 in its native environment – using MINFLUX nanoscopy^17–19^ and cryo-light microscopy^49^ – demonstrated that a large fraction of PIEZO1 channels ( ~ 30–40%) are captured in a flat conformation even in the absence of external mechanical stimulation. Considering that PIEZO1 only shows little spontaneous activity (e.g., induced by cell generated forces) in the absence of external mechanical stimulation, we hypothesised that these flat channels represent a flat inactivated conformation that might be stabilised by the docking of the wedge.

**Fig. 4:**
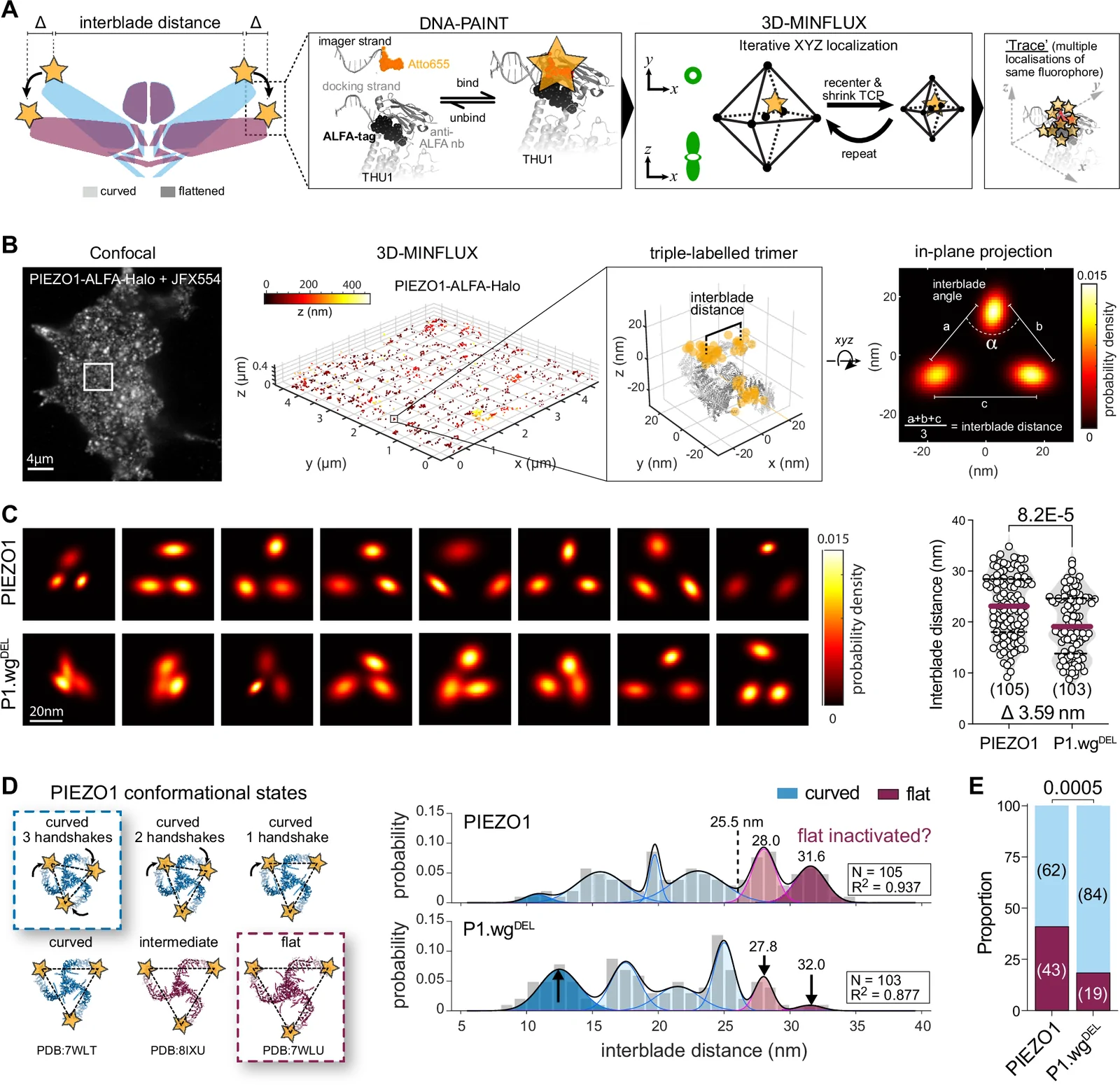
The wedge stabilises a flat inactivated conformation. **A** Cartoon depicting the overall strategy to resolve the PIEZO1 conformation in its native environment by measuring interblade distances. Insets (from left to right) illustrate the labelling of PIEZO1 with ALFA tag inserted after H86 and the DNA-PAINT method (left), the 3D-MINFLUX targeted coordinate pattern used for fluorophore position determination in 3D (middle) and an example of a MINFLUX ‘trace’ – i.e., a series of repeated localisations of the same fluorophore molecule. **B** Workflow example showing confocal image of a PIEZO1-ALFA-Halo expressing N2a-P1KO cell and labelled with JFX554 (left) and corresponding 3D-MINFLUX localisations (second from left), coloured by Z position. The inset shows a triple-labelled PIEZO1, with the traces (multiple localisations) of the three fluorophores superimposed on the cryo-EM structure. The 2D in-plane projections of the 3D data were fitted with a bivariate Gaussian distribution, with their probability densities, enabling determination of the average interblade distance (right). **C** In-plane projections of representative trimers examples of PIEZO1 (top left) and P1.wg^DEL^ (bottom left) together with a comparison of interblade distances (right) of PIEZO1 (*N* = 105) and P1.wg^DEL^ (*N* = 103) using a two-sided Mann-Whitney test (*P* = 8.2E-5). **D** Top views (left) of the indicated cryo-EM structures of possible closed (blue) and open (purple) states of PIEZO1. To enable depiction of interblade distances, the peripheral ends of the blade domains, which were not resolved by cryo-EM, were modelled by aligning the full-length Alphafold structure (AF-E2JF22) with cryo-EM structures. Domains originating from the Alphafold structure are shown as semi-transparent cartoons. To illustrate ‘handshaking’ between adjacent blades (top row), the peripheral blades were manually rotated counterclockwise. The right panel shows the interblade distance frequency distribution histograms of PIEZO1 (top) and P1.wg^DEL^ (bottom) fitted with a Gaussian mixture model with six components. Peaks of putative flat conformation are shown in purple and those of curved states in blue. **E** Comparison of the proportions of channels adopting flat (purple) and curved (blue) conformations using two-sided Fisher’s exact test (*P* = 0.0005). Numbers in brackets in panels (**C**–**E**) represent the number of biological replicates (i.e., number of triple labelled PIEZO1 trimers detected by MINFLUX). PIEZO1 cartoon adapted from [19] under CC BY 4.0 (https://creativecommons.org/licenses/by/4.0/).

To test this hypothesis, we utilised 3D-MINFLUX nanoscopy in combination with DNA-PAINT, which achieves spatial resolutions in the nanometre range^50–52^, thereby enabling us to resolve the distances between fluorophores attached to the peripheral ends of the blade domains – i.e., interblade distance (a surrogate measure for channel flattening) – and thus directly observe PIEZO1 conformation in its native environment (Fig. 4A, B). To this end, we inserted ALFA-tags^53^ into position H86 (extracellular distal end of the blade), which does not alter channel function^18,19^, and imaged cells with 3D-MINFLUX using a DNA-PAINT docking strand-conjugated single domain anti-ALFA nanobody and an Atto655-conjugated DNA-PAINT imager strand (Fig. 4A, B and Supplementary Fig. 5A). This labelling system has a linkage error of only ~ 2.5 nm, thereby allowing precise position estimates of the distal ends of the blades (Fig. 4A). Furthermore, the MINFLUX data obtained here had a localisation precision of less than 5 nm in all three dimension (Supplementary Fig. 5B). To identify triple-labelled PIEZO1 trimers in MINFLUX localisation data, we computed the Euclidian distance matrix of all localisations and searched for localisation triplets in which the distances between the individual localisations were smaller than 40 nm (i.e., the maximal physically possible distance between two ALFA-tags in the fully flattened state) and that had no other neighbouring signals within 60 nm. Since triple-labelled PIEZO1 trimers are relatively scarce, due to technical limitations of MINFLUX and limitations in labelling efficiency^17–19^, we also analysed the interblade distances of double-labelled trimers using the same cut-off criteria. Using this approach, we have previously shown that PIEZO1 conformation at rest varies between cellular compartments and is governed by cytoskeletal rigidity^18^ and have demonstrated Yoda-induced flattening of the channel^19^. In agreement with the work from Mazal and colleagues^49^ and our own previous work^18,19^, the interblade distance distribution of full-length PIEZO1 observed here was broad and non-uniform (Fig. 4C and D) and could be faithfully fitted with a Gaussian mixture model with six components (*N* = 105, R^2^ = 0.937), with four broad peaks below 25.5 nm, which likely represent curved conformations with different numbers of ‘handshakes’ between the peripheral blades^18,54^, and two distinct peaks at values greater than 25.5 nm, which possibly represent the intermediate^12^ and the fully flattened^13^ conformations, repsectively (Fig. 4D). Notably, the median interblade distance measured in triple-labelled trimers was significantly reduced by 3.59 nm in the P1.wg^DEL^ mutant as compared to full-length PIEZO1 (PIEZO1 = 23.2 ± 0.6 nm, *N* = 105 vs. P1.wg^DEL^ = 19.6 ± 0.6 nm, *N* = 103, *P* = 8.2E-5, Mann-Whitney test; Fig. 4C). Measurements of the interblade distances of double-labelled PIEZO1 trimers showed the same difference (Supplementary Fig. 5C) and the interblade angles were not altered by wedge deletion, indicating that the wedge only affects blade flattening but not rotational flexibility (Supplementary Fig. 5D). Detailed inspection of the interblade distance distribution histogram showed that the peak representing the fully flattened conformation was almost completely absent and the peak representing the intermediate flat conformation became significantly smaller in the interblade distance distribution of P1.wg^DEL^ (purple, Fig. 4D), whereas the leftmost peak, which possibly represents the curved conformation with three handshakes (dark blue, Fig. 4D), was significantly larger. To statistically compare the two distributions, we categorised all channels with interblade distances below 25.5 nm as curved and those with interblade distances greater than 25.5 nm as flat and preformed a Fisher’s exact test. This analysis revealed that the overall proportion of channels that adopt a flat state significantly decreases from 40.9 % to 18.4 % upon deletion of the wedge (P = 0.0005, Fisher’s exact test, Fig. 4E), suggesting that the wedge normally stabilises a flat inactivated conformation.

**Fig. 5:**
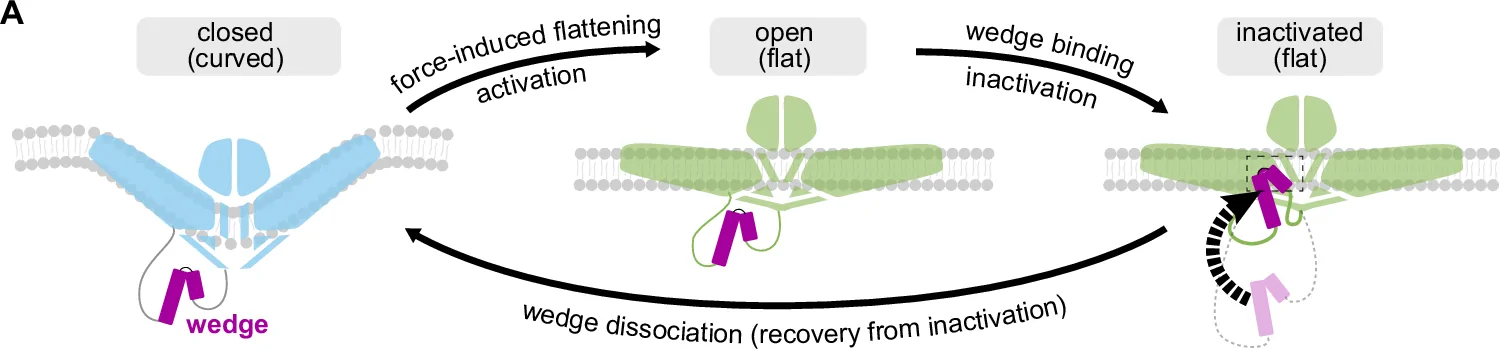
Mechanistic model of PIEZO1 inactivation via wedge docking. **A** Cartoon illustrating the mechanistic model of inactivation supported by our data. PIEZO1 cartoon adapted from [19] under CC BY 4.0 (https://creativecommons.org/licenses/by/4.0/).

In summary, previous cryo-EM data together with the electrophysiological and MINFLUX data obtained here support a ball-and-chain-like inactivation mechanism in which the wedge serves as an inactivation particle that docks to the anchor-OH-linker in a state-dependent manner (i.e., only in the flat conformation) to induce inactivation and stabilise a flat inactivated conformation (Fig. 5).

## Discussion

Inactivation is a common self-regulation mechanism of many ion channels, including voltage-gated sodium and potassium channels, some ligand-gated ion channels and not least PIEZO channels, which limits ion influx in the continuous presence of an activating stimulus and is thus essential for normal cellular function. In voltage-gated sodium and potassium channels, rapid inactivation is mediated by a mechanistically conserved ball-and-chain mechanism, where a small inactivation particle that is connected to the α-subunit of the channel via a flexible polypeptide chain docks to the pore or adjacent to the pore to terminate ion influx^55,56^. Here, we provide evidence suggesting that a similar ball-and-chain-like mechanism, where the wedge serves as the inactivation particle and the flexible linkers that connect the wedge to the clasp and the beam serve as the chain, contributes to the inactivation of PIEZO1 (Fig. 5). This inactivation model is supported by three key observations: firstly, the wedge has not been structurally resolved in any previously published cryo-EM structure of the curved (closed) PIEZO1 conformation^6–8^, whereas it was found docked to the lateral side of the pore module in the flat PIEZO1 conformation^13^ (Fig. 1A), which strongly suggests state-dependent wedge binding. Secondly, deletion of the wedge or disruption of specific non-covalent π-π and cation-π interactions between the wedge α1-helix and the anchor–OH-linker almost completely abolished inactivation in pressure-clamp recordings without otherwise impairing PIEZO1 function (Figs. 1C, D, F and G, 2C, D). Thirdly, measurements of PIEZO1 interblade distances – i.e., a surrogate measure for channel flattening – in the presence and absence of the wedge domain, using 3D-MINFLUX nanoscopy, indicate that the wedge is required to stabilise a flat and possibly inactivated conformation (Fig. 4).

While evidence for state-dependent binding of the wedge domain is solid and comes from three different laboratories that resolved the conformation of PIEZO1 in the curved^6–8^ and flat^13^ state using cryo-EM, the whereabouts of the wedge in the curved state and the choreography of its motions during inactivation are less clear (Fig. 1A). The secondary structure prediction algorithms PSIRPED, JNET and TRANSSEC^57–59^, together with the intrinsic disorder prediction tools IUPRED3 and PONDR(VL-XT)^60^ suggest that the wedge – irrespective of PIEZO1 conformation – adopts a stable secondary structure comprising two α-helices and that the linkers that attach the wedge to the clasp and beam are intrinsically disordered and thus probably highly flexible (Supplementary Fig. 1A, B). Moreover, Alphafold also assigns a defined secondary structure with two α-helices to the wedge domain in the curved state but places the wedge distant from the anchor–OH–linker where it cannot engage in non-covalent interactions with this region (AF-E2JF22)^61^. Hence, our electrophysiological characterisation of the point mutants that disrupt putative non-covalent interactions between the wedge and the anchor-OH-linker in the flat state (Fig. 2), together with the experimentally determined structures (Fig. 1A) and the in-silico predictions (Supplementary Fig. 1A and B), strongly suggest that the wedge, together with the flexible linkers form a ball-and-chain-like inactivation system that terminates ion flux during sustained mechanical stimulation by binding to the anchor-OH-linker. We have not examined the allosteric rearrangements that eventually lead to inactivation downstream of wedge docking and can thus only speculate about this mechanism. Previous studies have demonstrated a prominent role of the cap and the inner helix in PIEZO1 inactivation^9,10,37,38^. Hence, considering that the cap-OH spring linkers and the hydrophobic inactivation gate (L2475 and V2476), which are required for cap- and IH-dependent inactivation, respectively, are in close proximity to the wedge docking site, one possible explanation would be that wedge docking facilitates cap- and/or IH-dependent inactivation via allosteric coupling. It is, however, also possible that wedge-dependent inactivation represents a different and independent inactivation mechanism. Indeed, the wedge docking site – more generally the anchor-OH-linker – has previously been shown to be a crucial hub for the interaction with several PIEZO1 modulators that alter inactivation, including E-cadherin^40^, SERCA^41^ and PIP^36^. Moreover, recent cryo-EM structures complemented with functional patch-clamp data showed that the space between the anchor, OH and IH serves as a binding pocket for the auxiliary PIEZO1 subunit MDFIC^31^, which also controls channel inactivation and for a yet unidentified – possibly a lipid – cofactor that facilitates full channel flattening^62^. Thus, another possible mode-of-action of the wedge is that it directly competes with the afore-mentioned modulators or indirectly blocks access to their binding sites, such that they can exert stronger effects (i.e., slow inactivation) in the absence of the wedge.

A puzzling observation requiring further discussion is that wedge deletion appeared to impair the inactivation of poke-evoked currents less strongly than that of stretch-evoked currents. Two explanations may account for this apparent assay dependence.

First, poke and stretch stimuli likely activate PIEZO channels through partly distinct force-transmission pathways. This is best exemplified by the diametrically opposing effects of cytochalasin-D-induced disruption of the cytoskeleton, which potentiates stretch-evoked but diminishes poking-evoked current^2,18,45,63,64^. This has commonly been interpreted to indicate that PIEZO1 is activated predominantly by force-from-lipids, i.e., membrane-tension-dependent gating, in the pressure-clamp assay, whereas poke stimulation engages force-from-filament mechanisms, either directly or through tethering proteins. In addition, intra-molecular force coupling within PIEZO channels may differ between the two stimulus modalities. We previously identified an intracellular PIEZO2 domain required for activation by poke but not by stretch^64^, and Zhao et al. showed that mutations in residues proposed to couple the beam to the CTD, L1342 and L1345, nearly abolish poke-evoked PIEZO1 currents while reducing stretch-evoked currents by only approximately 50%^8^. Thus, if poke and stretch engage distinct cellular and intra-molecular activation pathways, they may also induce channel states with different inactivation requirements. In this scenario, the wedge domain may be preferentially required to inactivate the stretch-induced open state. Directly testing this possibility was beyond the scope of the present study.

A second, less speculative explanation is that decay of poke-evoked currents reflects a combination of inactivation and deactivation. Thus, even if inactivation is largely abolished, substantial current decay may persist during sustained indentation because the effective stimulus declines over time. This interpretation is supported by our AFM measurements showing rapid decay of indentation forces, most likely due to cell relaxation, and is consistent with previous AFM measurements in HEK293 cells^45^. Such stimulus adaptation could also explain why (i) poke-evoked PIEZO1 currents generally decay faster than stretch-evoked currents^1,43^, (ii) Yoda1-dependent slowing of inactivation is more pronounced in the pressure-clamp assay^19,65^ and (iii) in the few studies directly comparing both assays, mutations or auxiliary proteins consistently have smaller effects on current decay in the poking assay. The Cox lab, for example, showed that MDFIC co-expression essentially abolishes PIEZO1 inactivation in the pressure-clamp assay, with currents remaining close to peak amplitude throughout the stimulus, whereas poke-evoked currents still decayed to approximately 40% of peak amplitude^31^. Similarly, the Coste laboratory reported that MDFIC2 prevents inactivation of stretch-evoked PIEZO1 currents, while poke-evoked currents decayed to approximately 10% of peak amplitude^32^. Moreover, most disease-causing mutations also do not completely remove inactivation in the poking assay but merely slow it by 2-5 fold (e.g., R1943Q, M2225R, R2302H, R2456H, R2488Q – see Romero et al. for side-by-side comparison^35^), which is slightly higher or in the same range as the effect observed here. The only mutations that completely remove or strongly diminish current decay during sustained stimulation in the poking assay are those that also remove deactivation, such as the L2475/V2476/ M2493/F2494-QQQQ quadruple mutant described by Zheng et al.^10^ and the aforementioned auxiliary subunits MDFIC and MDFIC2^31–33^. Together, these observations support the conclusion that decay of poke-evoked PIEZO1 currents is not a pure measure of inactivation but is confounded by deactivation caused by relaxation of the mechanical stimulus, thereby highlighting an important limitation of the poking assay that should be considered in future studies.

We also examined the conformational changes involved in the recovery from inactivation using 3D-MINFLUX nanoscopy (Fig. 4). An intriguing observation from previous super-resolution imaging studies of PIEZO1 in its native environment by others and us^17–19,49^, was that an unexpectedly large fraction of PIEZO1 channels exist in the flattened conformation in the absence of mechanical stimulation, which is believed to be the main driving force for channel flattening. One possible explanation for this phenomenon is that PIEZO1 remains trapped in the flat conformation for quite some time after inactivation before returning to the curved conformation. Rare spontaneous activation events (e.g., triggered by cell-generated traction forces^66^) could thus cause accumulation of flat – yet inactivated – channels over time, even in the absence of exogenous mechanical stimulation, which could explain the large fraction of flat channels, although PIEZO1 only shows little background activity. Indeed, previous studies have shown that recovery from inactivation is very slow and may take up to ten seconds or longer^38,39^, and traction force-induced activation of PIEZO1 has also been elegantly demonstrated using advanced live-cell imaging^67^. Our 3D-MINFLUX data further supports this hypothesis, by showing that the interblade distribution of the full wedge deletion PIEZO1 mutant, which lacks inactivation (Fig. 1C, D) and immediately returns to a closed and “willing” state upon removal of the mechanical stimulus (Fig. 3), completely lacks the peak representing channels in the fully flattened conformation, while indicating that many more channels adopt a curved conformation (Fig. 4D, E), suggesting that wedge docking normally stabilises the flat conformation.

Hence, we propose a refined inactivation model for PIEZO1 where the wedge, in addition to the cap and inactivation gate of the inner helix, contributes to inactivation by binding to the anchor-OH-linker in a ball-and-chain-like manner. We further propose that wedge docking stabilises the flat inactivated conformation, and that dissociation of the wedge is required for the recovery from inactivation (Fig. 5).

By demonstrating that the wedge docks to a site that is enriched in binding sites for numerous PIEZO1 modulators (Fig. 2B) and considering that the wedge is encoded by a single exon (exon 32), the equivalent of which from PIEZO2 undergoes cell-type-specific splicing and slows inactivation^68^, our work also provides a framework for investigating the source of cell-type specific differences in PIEZO1 function that adjust its responsiveness to ensure optimal operation in different cellular environments.

## Methods

### Cell culture

Mouse neuroblastoma Neuro-2a PIEZO1-Knockout cell line (N2A-P1KO) was generated previously from Neuro-2a ATCC CCL-131 (a gift from G.R. Lewin XX). Cell culture conditions were 37 °C atmosphere with 5% CO2 in a 1:1 mixture of Dulbecco’s Modified Eagle Medium and Optimal Minimal Essential Medium, with 10% Fetal Bovine Serum, 2 mM L-glutamine and 1% peniciline/streptomycine (all purchased from ThermoFisher). Seeding was performed on methanol- and acid-washed glass coverslips and coated with poly-Llysine (PLL, Sigma). 12 mm diameter coverslips were used for patch clamp recordings and calcium imaging, and #1.5 and 18 mm diameter for Minflux imaging. Tranfection was performed 24 to 48 hs after seeding with polyethylenimine (PEI, Linear PEI 25 K, Polysciences). For each mL of culture medium 7 µL with 29 µL of PBS and 0.6 µg of Plasmid DNA, and incubated for 10 to 15 min at room temperature. The solution is then added to the culture medium and mixed gently by swirling. When preparing for calcium imaging experiments, 0.3 µg/mL medium of jGCaMP8m is added as well. After incubation for 24 h, the medium is exchanged for a fresh one, and the cells are used after at least 24 and no later than 48 h. Transfected cells were labelled with 1 nM JFX554 (Promega) diluted in culture medium, incubated overnight before the experiments.

### Interdomain interaction predition

Prediction of interdomain interactions was performed by the RING4.0 algorithm^46^. The wedge as well as surrounding structures of the flat PIEZO1 (PDB: 7WLU) was used. The predicted interactions were then sorted by quality of interaction, and bonds stronger than Van der Waals interactions were chosen.

### Structure modelling and visualisation

A full-length PIEZO1 was generated of the flat (PDB: 7WLU) and the curved (PDBs: 6BPZ, 6B3R, 5Z10) structures, which were aligned and superimposed for visualisation purposes. All modification operations and visualisation were performed in PyMol (Schrodinger). Plasmid design and visualisation was performed with SnapGene (version 7, Dotmatics). All the other data, graphics and schematics were elaborated and visualised in Matlab, IgorPro, Illustrator (Adobe) and GraphPad Prism (version 10, GraphPad Software).

### Generation of PIEZO mutants

PIEZO1-ALFA (instertion if ALFA-tag after H86) plasmids, which are expressing mutated mouse PIEZO1 and were previously shown not to affect PIEZO1 function^19^, were used as the template to generate firstly the PIEZO1-ALFA-Halo (insertion of Halo-tag at the C-terminus). From this template all other mutants used in this work were generated. Point mutants were introduced by site directed mutagenesis (primers were purchased from Sigma, sequence, template and obtained constructs can be found in Supplementary Table 1). The PCR reactions were digested with Dpln (New England Biolabs, 37 °C, 1 h) and column purified with standard kits (PureLink from Invitrogen or NucleoSpin from Macherey-Nagel) before transformation in electrocompetent Dh5a bacteria (Invitrogen) and grown at 37 °C overnight. Deletions were generated by Gibson cloning.

### Electrophysiology

PIEZO1 mutants were characterised using twofold patch-clamp assays either stimulating the cell via indentation (poking, whole-cell) or applying negative pressure pulses to the patch (stretch, cell-attach). PIEZO1-ALFA-Halo served as the control in all experiments. All recordings were acquired using room temperature buffers that were adapted for each use (see below). An EPC10 amplifier with Patchmaster software (HEKA Elektronik) was used for all recordings. The pipettes were pulled with a P-97 Flaming-Brown puller (Sutter Instrument) from borosilicate pipettes (Sutter instruments) (2–6 MΩ for whole-cell, 1.5–4.5 MΩ after fire-polishing for cell-attach). Cells were held at a holding potential of −60 mV in both assays. The responses to mechanical stimulation were recorded with a sampling frequency of 50 kHz and a filter with a 2.9 kHz low pass filter. For whole-cell patch-clamp recordings the intracellular buffer contained: 125 mM K-gluconate, 7 mM KCl, 1 mM MgCl2, 1 mM CaCl2, 4 mM EGTA, 10 mM HEPES (pH 7.3 with KOH). The bath solution contained: 140 mM NaCl, 4 mM KCl, 1 mM MgCl2, 2 mM CaCl2, 4 mM glucose and 10 mM HEPES (pH 7.4 with NaOH). Mechanical stimulation was applied by poking the cells with a firepolished glass pipette (tip diameter 2–3 μm) that was positioned opposite to the recording pipette, at an angle of approximately 45° to the surface. The cells were stimulated in series of 15 stimuli with increments of 0.8 µm. The poking pipette was positioned and moved during the stimulation using a piezo driven micromanipulator (Preloaded Piezo actuator P-840.20, Physik Instrumente) with a speed of 1 μm/ms. For cell-attached pressure-clamp recording the intracellular buffer contained: 130 mM NaCl, 5 mM KCl, 1 mM MgCl2, 1 mM CaCl2, 10 mM HEPES, 10 mM TEA-Cl (pH7.3 with NaOH). The bath solution contained: 140 mM KCl, 1 mM MgCl2, 2 CaCl2, 10 Glucose, 10 HEPES (pH7.4 with KOH). Gigaseal was established using the High-Speed Pressure Clamp device (HSPC; ALA Scientific Instruments) with a brief −10mmHg pulse. The patch was stimulated by lowering the intrapipette pressure for 500 ms per stimulus in series of 16 increments of − 5 mmHg. A prepulse + 5 mmHg was applied for 1 s before the stimulus to improve recovery from inactivation^39^. For repeated stimulations the cells were stimulated in a series of 6 pulses, each lasting 250 ms with − 60 mmHg and + 5 mmHg applied in the inter-pulse intervals. The duration of the inter-pulse intervals was varied between series applied consecutively to the same patch, starting at 1000 ms and reducing the duration of by half until 50 ms were applied in the last series of six pulses. For prepulse-conditioned measurements, the baseline amplitude was measured with a pulse of 500 ms at − 60 mmHg following 500 ms at +5 mmHg, then holding the cell at 0 mmHg for 5 s to ensure full recovery and then giving a prepulse of − 20 mV for 500 ms before again stimulating with − 60 mmHg. For the acquisition of I/V and single-channel conductance experiments, the stimulation pressure was adjusted for the individual cell to optimise for the evocation of single-channel openings. The conductance was determined by applying holding potentials from − 140 mV to − 40 mV in increments of 20 mV.

All electrophysiology was analysed using custom scripts in IgorPro (Wavemetrics). For poking-evoked currents, mechanical activation thresholds were defined as the stimulus that evoked the first peak current that was more than six standard deviations of the baseline above the baseline. The inactivation time constants (τinact) were measured by fitting the mechanically activated currents with a single exponential function (C1 + C2*e (–(t–t0)/τinact), where C1 and C2 are constants, t is time, and τinact is the inactivation time constant (only peak currents between 100 and 1500 pA were used for these calculations and averaged per cell.

Peak/sustained ratios shown in Figs. 1, 2 are averages of the ratios from three pressure pulses (i.e., − 50, − 55 and − 60 mmHg).

The peaks of pressure-induced currents (I) were normalised to the absolute maximal response of the cell at any pressure (Imax). Normalised pressure-response curve (I/Imax) from individual cells were fitted with a Boltzmann sigmoid to determine individual P50 (in mmHg).

For repeated stimulations, the peak of the ratio of the first peak and every subsequent peak (I/I_stim#1_) was calculated. When conditioning the cell with a prepulse, the ratio of the conditioned and unconditioned peak of the response to the − 60 mmHg stimulation was calculated (I_+pre_/I_-pre_).

Single-channel amplitudes at a given holding potential ( − 140 mV to −40 mV, 20 mV steps) were determined as the difference between the peaks of the Gaussian fits of the trace histogram over multiple 1 s segments. Unitary conductance was determined from the linear regression fits of the I/V plot of individual cells. Recordings with excessive leak currents or unstable baselines were excluded. Recordings that displayed non-inactivating responses or unstable openings were also not used for further I/V analyses.

### Calcium imaging

N2A-P1KO cells were cultured and transfected as described above. 48 to 72 h after transfection the cells were washed once with PBS and incubated with a calcium imaging buffer containing 140 mM NaCl, 4 mM KCl, 1 mM MgCl2, 3 mM CaCl2, 4 mM glucose and 10 mM HEPES (pH 7.4 with NaOH). Fluorescent images were acquired every two seconds (500 ms exposure time) on an Olympus BX40 upright microscope equipped with a standard Quad filter (Chroma), fluorescent lamp (HBO 100) and shutter (Lambda 10-2, Sutter Instrument) with a 20 × water-immersion objective (UMPLFLN 20XW, Olympus), visualised with a Kiralux CS895MU camera (Thorlabs) and acquired with MicroManager^69^. For perfusion and fast solution exchange a gravity-driven perfusion system (ValveLink8.2, AutoMate Scientific). The cell perfusion protocol was as follows: 30 s wash with buffer, 30 s perfusion with Yoda2 solution (Tocris, 1 nM, 10 nM, 100 nM, 1 µM, 10 µM), 240 s wash with buffer. Two fields of view were acquired per coverslip, moving for the second field of view upstream from the first, always using low Yoda2 concentrations (1 nM, 10 nM) as first and high Yoda2 concentrations (100 nM, 1 µM, 10 µM) as second stimulation to avoid desensitisation.

To segment the cells and measure the fluorescence intensities a custom macro in ImageJ FIJI was used. The ratio of the fluorescence and the baseline (mean fluorescence of the first 15 s of the initial wash with buffer) was calculated over time (F/F_0_). Within the Yoda2 perfusion time, the maximal F/F_0_ per cell was extracted and fitted with a sigmoid curve for EC_50_ calculation.

### Confocal imaging

N2A-P1KO cells were plated on #1.5 12 mm coverslips and transfected with PIEZO1-ALFA-Halo or associated wedge-deletion mutants. 48 to 72 h after transfection, cells were washed once with PBS and fixed with paraformaldehyde (1%) and glutaraldehyde (0.05%) for 20 min at room temperature. Fixatives were quenched with 5 mM NaBH4 in PBS and then with 50 mM glycine and 50 mM NH4Cl in PBS. Cells were further washed thrice with PBS and blocked with BSA 2% in PBS for 1 h at room temperature. As the ALFA tag is located on the extracellular side, no permeabilisation was performed. Labelling was done with FluoTag X2 Anti ALFA conjugated to Alexa647 (NanoTag, RRID:AB_3075981) diluted 1:100 in PBS + BSA 2% and incubated overnight at 4 °C. Samples were washed with PBS and mounted on a slide with ProLong Diamond antifade with Dapi (Invitrogen). Confocal images were acquired on a Zeiss Axio Examiner Z1 equipped with an LSM 980 and Airyscan 2 module (Zeiss). A 63x oil Plan-APOCHROMAT objective was used together with continuous wave lasers (405 for Dapi and 639 for Alexa647) for excitation and the Airyscan 2 array detector. Images were taken at the equatorial plane of the cells. The Zeiss ZEN software was used for image acquisition.

### Atomic force microscopy

The temporal dynamics of indentation forces were measured in live N2a cells using an atomic force microscope (Nanowizard I, JPK Instruments, Germany) equipped with a tipless gold-coated cantilever with a spring constant of 0.2407 N/m (MLCT-O10, Bruker). Cells were washed with patch-clamp buffer and mounted on the AFM. Prior to cell stimulation, photodetector sensitivity was calibrated by pressing the cantilever against the bare glass using the inverse optical lever sensitivity (invOLS) method^70^. To measure indentation force dynamics, cell were compressed by moving the cantilever towards the cell with constant velocity (0.0025 µm/ms, 0.005 µm/ms, 0.01 µm/ms, 0.02 µm/ms, 0.05 µm/ms and 0.1 µm/ms were tested; see Fig. 1I), while the force acting on the cantilever was registered at a sampling rate of 2048 Hz in contact mode. Each indentation velocity was tested at least 5 times per cell at an interval of 5 s. Average values of individual cells are reported in the source data file provided with this manuscript the total averages of all tested cells are shown in Fig. 1I.

### Preparation of samples for 3D-MINFLUX/DNA-PAINT imaging

N2A-P1KO cells were plated on #1.5 18 mm coverslips, transfected and labelled as described above; the cells were prepared for imaging 48 to 72 h after transfection. Preparation started by fixing the cells in paraformaldehyde (1%) and glutaraldehyde (0.05%) for 20 min at room temperature, and quenched for 10 min with 5 mM NaBH4 and then with a mix of 50 mM glycine and 50 mM NH4Cl, all diluted in PBS, and then further washed with PBS. Samples were blocked with Antibody Incubation buffer (Massive Photonics) for 30 min at room temperature. The cells were incubated for 1 h at room temperature or overnight at 4 °C with Massive-Tag-Q Anti-ALFA nanobodies conjugated with a DNA docking strand (both from Massive Photonics) diluted at 1:100 (Anti-ALFA) in Antibody Incubation buffer. After washing thrice with 1 × Washing buffer (Massive Photonics), the cells were post-fixed for 5 min, and the fixatives were quenched and washed as described above. Samples were then incubated for 10 min with 200 μl of gold nanoparticles for future stabilisation (gold colloid 250 nm, BBI Solutions). The cells were washed several times with PBS to remove excess unbound nanoparticles, and remaining nanoparticles were stabilised by incubating the sample with PLL for 60 min at room temperature. Cells were again washed thrice with PBS before mounting and used over the course of the following two days. Corresponding DNA-PAINT imagers (Massive Photonics) conjugated to Atto 655 were freshly diluted in Imaging buffer (Massive Photonics) for a final concentration of 1 ~ 2 nM (Atto 655, Imager sequence #3). A drop of imager dilution was added into a cavity slide, and coverslips were mounted and sealed with Picodent Twinsil (Picodent).

### 3D-Minflux/DNA-PAINT imaging

To localise individual fluorophore molecules, MINFLUX microscopy, like other super-resolution techniques, requires that only a single fluorophore within the volume that is illuminated by the excitation laser emits light at a given time, which is usually achieved by using fluorophores that ‘blink’ – i.e., randomly switch between a bright and a dark state – under certain experimental conditions. Here, we used an alternative approach called DNA-PAINT, which utilises complementary pairs of short oligonucleotides that transiently bind to each other. One oligonucleotide, denoted as the docking strand, is conjugated to a single domain nanobody that recognises the target protein, while the second oligonucleotide – the imager strand – is fluorescently labelled and freely diffuses in the mounting medium, such that transient binding of the oligonucleotide pairs produces blinking-like behaviour. To determine the position of individual fluorophores, the MINFLUX microscope performs an iterative 3D TCP (targeted coordinate pattern) scanning procedure, which is repeated until the fluorophore is bleached or the imager strand dissociates from the target, such that ‘traces’ with multiple localisation estimates of the same fluorophore are generated (Fig. 4A). In each iteration the TCP is shrunk and re-centred to the putative position of the fluorophore, which is estimated by triangulation based on the photon counts in the individual scan positions. To rule out confounding signals from multiple fluorophores in the scan region and otherwise poorly localised fluorophores, the iterative scan is terminated when the CFR (centre frequency ratio) – i.e., the ratio of the photon emission frequency at the centre position of the MINFLUX excitation pattern over the mean effective emission frequency over all outer positions – exceeds 0.8 in the fourth iteration step (Supplementary Table 2). An Abberior MINFLUX commercial microscope built on an inverted IX83 microscope with a 100× UPlanXApo objective (Olympus) and using Imspector Software (Abberior Instruments) was used to acquire all Minflux data. The calibration and alignment of the excitation beam pattern, as well as the pinhole positioning, were performed at the beginning of each acquisition session using fluorescent nanoparticles (Abberior Nanoparticles, Gold 150 nm, 2 C Fluor 120 nm, Abberior Instruments). Cells transfected with PIEZO-ALFA-Halo were identified with a 561 nm confocal scan. The focus was set to the interface between the coverslip and the cell membrane. At least 2 gold fiducials were present in the field of view and used by the active feedback stabilisation system of the microscope (IR 975 nm laser, Cobolt, and CCD camera, The Imaging Source), resulting in a precision below 1 nm in all three axes and being stabled for hours. Additional drift correction was performed actively by the system during acquisition, with the beamline monitoring feature. A ROI of a ~ 3 × 3 to 5 × 5 μm (and up to 8 × 8 μm for some overnight recordings) was selected. Laser power in the first iteration was set at 16% laser power, and the pinhole was set to 0.83 A.U. Final laser power in the last iteration is scaled up by a factor of six. ROIs were imaged for at least 2 h and up to overnight (~ 12 h) using the manufacturers default 3D Minflux TCP sequence. Detection for Atto-655 signals was performed with two avalanche photodiode channels (650–685 nm and 685–720 nm) that were pooled.

### 3D-Minflux data analysis

Valid Minflux localisation data from the final iterations was exported as MATLAB files from the Imspector software and analysed using costum matlab scripts for post-processing and filtering, further operations and data visualization was also performed in Matlab. The data was filtered in a stepped process firstly by cfr (centre frequency ratio, directly implemented during the measurement, set at 0.8) and efo (effective frequency at offset, kHz, retrieved for each individual valid locations) to dismiss potential multiple emitters. Secondly, localisations from the same emission trace (ones sharing one trace identification number (TID)), having a standard deviation higher than 10 nm in any dimension and such that contained less than 3 localisations were filtered-out. For each trace, standard deviations were calculated separately for x, y and z coordinates using the standard formula. The first two localisations of each trace were discarded, since they tend to deviate from the majority of the cloud. A scaling factor of 0.7 was applied to all z-coordinates to correct for the refractory index mismatch between the coverslip and sample^50^. The geometric centre for each individual filtered trace was then calculated. Due to the nature of DNA-PAINT, different traces originating from repeated detection of the same PIEZO protomer were identified using DBSCAN clustering with minPoints of 2 and epsilon of 8 nm. This cutoff was selected based on the localisation precision of MINFLUX and the possible ALFA-tag flexibility^18^. Protomers that were detected several times were represented by the mean coordinate of the localisations clustered by this DBSCAN algorithm. Lastly PIEZO1 trimers that were a) fully labelled and b) fully detected by Minflux were determined by searching the cloud of traces for clusters of three adjacent traces with distances to each other that were smaller than 40 nm (the assumed maximal distance two Atto-655 molecules bound to the same PIEZO1 trimer can possibly have based on available flattened PIEZO1^18^ and distances to the next closest trace of over 60 nm. Only trimers with a maximum internal angle of less than 120° were included. The mean interblade distance for each trimer was calculated. Further visualisation of trimers was done by a 2D in-plane projection of the raw localisations for each protomer, followed by a fit with a bivariate Gaussian distribution and displayed with its probability density.

### Statistical tests and reproducibility

All experiments in this study were performed independently at least three times, yielding similar results. For Minflux experiments, data are from at least 4 cells from at least 3 independent experiments. No statistical method was used to predetermine sample size. Experiments were not randomised, and investigators were not blinded during experiments and analysis. Data distribution was systematically evaluated using D’Agostino–Pearson test, and parametric or nonparametric tests were chosen accordingly. The statistical tests that were used, the exact P-values and information about the number of replicates are provided in the figure or the corresponding legends.

### Reporting summary

Further information on research design is available in the Nature Portfolio Reporting Summary linked to this article.

## Supplementary information

Supplementary Information

Reporting Summary

Transparent Peer Review file

## Source data

Source Data

## Data Availability

The MINFLUX raw data generated in this study and corresponding confocal images have been deposited together with the custom written Matlab analysis code on Github [https://github.com/StefanLechnerUKE/PIEZO1_wedge_analysis] and at Zenodo [10.5281/zenodo.21363175]^71^. The patch-clamp, calcium imaging and AFM data generated in this study are provided in the Source Data File. Previously published PDB files that were used for wedge interaction analysis are available at: 7WLT, 7WLU, 6B3R, 5Z10, 6BPZ. Source data are provided in this paper.

## Code availability

The custom Matlab codes for Minflux analysis are available at https://github.com/StefanLechnerUKE/PIEZO1_wedge_analysis and at Zenodo [10.5281/zenodo.21363175]^71^.

## References

[1] Coste, B. et al. Piezo1 and Piezo2 are essential components of distinct mechanically activated cation channels. *Science* **330**, 55–60 (2010). 20813920 10.1126/science.1193270 PMC3062430

[2] Gottlieb, P. A., Bae, C. & Sachs, F. Gating the mechanical channel Piezo1: a comparison between whole-cell and patch recording. *Channels* **6**, 282–289 (2012). 22790451 10.4161/chan.21064 PMC3508907

[3] Kefauver, J. M., Ward, A. B. & Patapoutian, A. Discoveries in structure and physiology of mechanically activated ion channels. *Nature* **587**, 567–576 (2020). 33239794 10.1038/s41586-020-2933-1 PMC8477435

[4] Wu, J., Lewis, A. H. & Grandl, J. Touch, Tension, and Transduction – The Function and Regulation of Piezo Ion Channels. *Trends Biochem. Sci.* **42**, 57–71 (2017). 27743844 10.1016/j.tibs.2016.09.004 PMC5407468

[5] Xiao, B. Mechanisms of mechanotransduction and physiological roles of PIEZO channels. *Nat. Rev. Mol. Cell Biol.* **25**, 886–903 (2024). 39251883 10.1038/s41580-024-00773-5

[6] Saotome, K. et al. Structure of the mechanically activated ion channel Piezo1. *Nature*10.1038/nature25453 (2017). 29261642 10.1038/nature25453 PMC6010196

[7] Guo, Y. R. & MacKinnon, R. Structure-based membrane dome mechanism for Piezo mechanosensitivity. *ELife* **6**, e33660 (2017). 29231809 10.7554/eLife.33660 PMC5788504

[8] Zhao, Q. et al. Structure and mechanogating mechanism of the Piezo1 channel. *Nature*10.1038/nature25743 (2018). 29469092 10.1038/nature25743

[9] Liu, W. et al. Spring-like mechanics enable rapid inactivation and stochastic single-channel gating of the mechanically activated PIEZO channel. *Cell Rep.* **44**, 116615 (2025). 41307990 10.1016/j.celrep.2025.116615

[10] Zheng, W., Gracheva, E.O., & Bagriantsev, S.N. A hydrophobic gate in the inner pore helix is the major determinant of inactivation in mechanosensitive Piezo channels. Elife** 8**, 10.7554/eLife.44003 (2019). 10.7554/eLife.44003 PMC6349400 30628892

[11] Geng, J. et al. A plug-and-latch mechanism for gating the mechanosensitive piezo channel. *Neuron* **106**, 438–451 (2020). 32142647 10.1016/j.neuron.2020.02.010

[12] Liu, S. et al. An intermediate open structure reveals the gating transition of the mechanically activated PIEZO1 channel. *Neuron*10.1016/j.neuron.2024.11.020 (2024). 39719701 10.1016/j.neuron.2024.11.020

[13] Yang, X. et al. Structure deformation and curvature sensing of PIEZO1 in lipid membranes. *Nature* **604**, 1–7 (2022). 10.1038/s41586-022-04574-8 35388220

[14] Haselwandter, C. A., Guo, Y. R., Fu, Z. & MacKinnon, R. Quantitative prediction and measurement of Piezo’s membrane footprint. *Proc. Natl. Acad. Sci. USA* **119**, e2208027119 (2022). 36166475 10.1073/pnas.2208027119 PMC9546538

[15] Haselwandter, C. A. & MacKinnon, R. Piezo’s membrane footprint and its contribution to mechanosensitivity. *ELife* **7**, e41968 (2018). 30480546 10.7554/eLife.41968 PMC6317911

[16] Lin, Y.-C. et al. Force-induced conformational changes in PIEZO1. *Nature* **573**, 230–234 (2019). 31435018 10.1038/s41586-019-1499-2 PMC7258172

[17] Mulhall, E. M. et al. Direct observation of the conformational states of PIEZO1. *Nature* **620**, 1117–1125 (2023). 37587339 10.1038/s41586-023-06427-4 PMC10468401

[18] Verkest, C. et al. Cluster nanoarchitecture and structural diversity of PIEZO1 at rest and during activation in intact cells. *Sci. Adv.* **11**, eady8052 (2025). 41124252 10.1126/sciadv.ady8052 PMC12542951

[19] Verkest, C., Roettger, L., Zeitzschel, N. & Lechner, S. G. 3D-MINFLUX nanoscopy reveals distinct allosteric mechanisms for activation and modulation of PIEZO1 by Yoda1. *Nat. Commun.*10.1038/s41467-025-67610-x (2025). 41402296 10.1038/s41467-025-67610-x PMC12712055

[20] Wang, Y. et al. A lever-like transduction pathway for long-distance chemical- and mechano-gating of the mechanosensitive Piezo1 channel. *Nat. Commun.* **9**, 1300 (2018). 29610524 10.1038/s41467-018-03570-9 PMC5880808

[21] Taberner, F. J. et al. Structure-guided examination of the mechanogating mechanism of PIEZO2. *Proc. Natl. Acad. Sci. USA* **116**, 14260–14269 (2019). 31235572 10.1073/pnas.1905985116 PMC6628815

[22] Vero Li, J., D Cox, C. & Martinac, B. The anchor domain is critical for Piezo1 channel mechanosensitivity. *Channels* **15**, 438–446 (2021). 33975519 10.1080/19336950.2021.1923199 PMC8118467

[23] Wu, J., Goyal, R. & Grandl, J. Localized force application reveals mechanically sensitive domains of Piezo1. *Nat. Commun.* **7**, 12939 (2016). 27694883 10.1038/ncomms12939 PMC5063965

[24] Zarychanski, R. et al. Mutations in the mechanotransduction protein PIEZO1 are associated with hereditary xerocytosis. *Blood* **120**, 1908–1915 (2012). 22529292 10.1182/blood-2012-04-422253 PMC3448561

[25] Bae, C., Gnanasambandam, R., Nicolai, C., Sachs, F. & Gottlieb, P. A. Xerocytosis is caused by mutations that alter the kinetics of the mechanosensitive channel PIEZO1. *PNAS* **110**, E1162–E1168 (2013). 23487776 10.1073/pnas.1219777110 PMC3606986

[26] Glogowska, E. et al. Novel mechanisms of PIEZO1 dysfunction in hereditary xerocytosis. *Blood* **130**, 1845–1856 (2017). 28716860 10.1182/blood-2017-05-786004 PMC5649553

[27] Albuisson, J. et al. Dehydrated hereditary stomatocytosis linked to gain-of-function mutations in mechanically activated PIEZO1 ion channels. *Nat. Commun.* **4**, 1884 (2013). 23695678 10.1038/ncomms2899 PMC3674779

[28] Alper, S.L. in Current Topics in Membranes Piezo Channels. (Academic Press, 2017).

[29] Anderson, E. O., Schneider, E. R., Matson, J. D., Gracheva, E. O. & Bagriantsev, S. N. TMEM150C/tentonin3 is a regulator of mechano-gated ion channels. *Cell Rep.* **23**, 701–708 (2018). 29669276 10.1016/j.celrep.2018.03.094 PMC5929159

[30] Koster, A. K. et al. Chemical mapping of the surface interactome of PIEZO1 identifies CADM1 as a modulator of channel inactivation. *Proc. Natl. Acad. Sci. USA* **121**, e2415934121 (2024). 39356664 10.1073/pnas.2415934121 PMC11474052

[31] Zhou, Z. et al. MyoD-family inhibitor proteins act as auxiliary subunits of Piezo channels. *Science* **381**, 799–804 (2023). 37590348 10.1126/science.adh8190

[32] Habib, A. M. et al. MDFIC2 is a PIEZO channel modulator that can alleviate mechanical allodynia associated with neuropathic pain. *Proc. Natl. Acad. Sci. USA* **122**, e2512426122 (2025). 41201821 10.1073/pnas.2512426122 PMC12626003

[33] Zhou, Z. et al. MDFIC2 is a sensory neuron–specific PIEZO channel auxiliary subunit. *Proc. Natl. Acad. Sci. USA* **123**, e2530071123 (2026). 41955114 10.1073/pnas.2530071123 PMC13080014

[34] Shi, J. et al. Sphingomyelinase disables inactivation in endogenous PIEZO1 channels. *Cell Rep.* **33**, 108225 (2020). 33027663 10.1016/j.celrep.2020.108225 PMC7539531

[35] Romero, L. O. et al. Dietary fatty acids fine-tune Piezo1 mechanical response. *Nat. Commun.* **10**, 1200 (2019). 30867417 10.1038/s41467-019-09055-7 PMC6416271

[36] Buyan, A. et al. Piezo1 forms specific, functionally important interactions with phosphoinositides, cholesterol and other lipid types. *Biophys. J.*10.1016/j.bpj.2020.07.043 (2020). 32949489 10.1016/j.bpj.2020.07.043 PMC7642233

[37] Lewis, A. H. & Grandl, J. Inactivation kinetics and mechanical gating of piezo1 Ion channels depend on subdomains within the cap. *Cell Rep.* **30**, 870–880 (2020). 31968259 10.1016/j.celrep.2019.12.040 PMC7021530

[38] Wu, J. et al. Inactivation of mechanically activated piezo1 ion channels is determined by the C-terminal extracellular domain and the inner pore helix. *Cell Rep.* **21**, 2357–2366 (2017). 29186675 10.1016/j.celrep.2017.10.120 PMC5938756

[39] Lewis, A. H., Cui, A. F., McDonald, M. F. & Grandl, J. Transduction of repetitive mechanical stimuli by piezo1 and piezo2 ion channels. *Cell Rep.* **19**, 2572–2585 (2017). 28636944 10.1016/j.celrep.2017.05.079 PMC5646378

[40] Wang, J. et al. Tethering Piezo channels to the actin cytoskeleton for mechanogating via the cadherin-β-catenin mechanotransduction complex. Cell Rep.** 38**, 10.1016/j.celrep.2022.110342 (2022). 10.1016/j.celrep.2022.110342 35139384

[41] Zhang, T., Chi, S., Jiang, F., Zhao, Q. & Xiao, B. A protein interaction mechanism for suppressing the mechanosensitive Piezo channels. *Nat. Commun.* **8**, 1797 (2017). 29176668 10.1038/s41467-017-01712-z PMC5702604

[42] Moroni, M., Servin-Vences, M. R., Fleischer, R., Sánchez-Carranza, O. & Lewin, G. R. Voltage gating of mechanosensitive PIEZO channels. *Nat. Commun.* **9**, 1096 (2018). 29545531 10.1038/s41467-018-03502-7 PMC5854696

[43] Zeitzschel, N. & Lechner, S. G. The activation thresholds and inactivation kinetics of poking-evoked PIEZO1 and PIEZO2 currents are sensitive to subtle variations in mechanical stimulation parameters. *Channels* **18**, 2355123 (2024). 38754025 10.1080/19336950.2024.2355123 PMC11734767

[44] Lewis, A. mandaH. & Grandl, J. Stretch and poke stimulation for characterizing mechanically activated ion channels. *Methods Enzymol.* **654**, 225–253 (2021). 34120715 10.1016/bs.mie.2020.12.024

[45] Young, M. N., Sindoni, M. J., Lewis, A. H., Zauscher, S. & Grandl, J. The energetics of rapid cellular mechanotransduction. *Proc. Natl. Acad. Sci. USA* **120**, e2215747120 (2023). 36795747 10.1073/pnas.2215747120 PMC9974467

[46] Del Conte, A. et al. RING 4.0: faster residue interaction networks with novel interaction types across over 35,000 different chemical structures. *Nucleic Acids Res.* **52**, W306–W312 (2024). 38686797 10.1093/nar/gkae337 PMC11223866

[47] Slavchov, R. I., Nomura, T., Martinac, B., Sokabe, M. & Sachs, F. Gigaseal mechanics: creep of the gigaseal under the action of pressure, adhesion, and voltage. *J. Phys. Chem. B* **118**, 12660–12672 (2014). 25295693 10.1021/jp506965v PMC4226309

[48] Wang, H. J. et al. Microscale geometrical modulation of PIEZO1 mediated mechanosensing through cytoskeletal redistribution. *Nat. Commun.* **15**, 5521 (2024). 38951553 10.1038/s41467-024-49833-6 PMC11217425

[49] Mazal, H., Wieser, F.-F., Bollschweiler, D., Schambony, A. & Sandoghdar, V. Cryo-light microscopy with angstrom precision deciphers structural conformations of PIEZO1 in its native state. *Sci. Adv.* **11**, eadw4402 (2025). 40834076 10.1126/sciadv.adw4402 PMC12366687

[50] Gwosch, K. C. et al. MINFLUX nanoscopy delivers 3D multicolor nanometer resolution in cells. *Nat. Methods* **17**, 217–224 (2020). 31932776 10.1038/s41592-019-0688-0

[51] Balzarotti, F. et al. Nanometer resolution imaging and tracking of fluorescent molecules with minimal photon fluxes. *Science* **355**, 606–612 (2017). 28008086 10.1126/science.aak9913

[52] Ostersehlt, L. M. et al. DNA-PAINT MINFLUX nanoscopy. *Nat. Methods* **19**, 1072–1075 (2022). 36050490 10.1038/s41592-022-01577-1 PMC9467913

[53] Götzke, H. et al. The ALFA-tag is a highly versatile tool for nanobody-based bioscience applications. *Nat. Commun.* **10**, 4403 (2019). 31562305 10.1038/s41467-019-12301-7 PMC6764986

[54] Smith, K. A. et al. Regulation of PIEZO1 channel force sensitivity by interblade handshaking. *Sci. Adv.* **11**, eadt7046 (2025). 40512861 10.1126/sciadv.adt7046 PMC12164982

[55] Liu, Y., Galpin, J. D., Ahern, C. A. & Bezanilla, F. Molecular basis of sodium channel inactivation. *Nat. Commun.* **16**, 10565 (2025). 41298396 10.1038/s41467-025-65587-1 PMC12658180

[56] Sukomon, N., Fan, C. & Nimigean, C. M. Ball-and-Chain Inactivation in Potassium Channels. *Annu. Rev. Biophys.* **52**, 91–111 (2023). 36626766 10.1146/annurev-biophys-100322-072921

[57] Montgomerie, S. et al. PROTEUS2: a web server for comprehensive protein structure prediction and structure-based annotation. *Nucleic Acids Res.* **36**, W202–W209 (2008). 18483082 10.1093/nar/gkn255 PMC2447806

[58] Jones, D. T. Protein secondary structure prediction based on position-specific scoring matrices. *J. Mol. Biol.* **292**, 195–202 (1999). 10493868 10.1006/jmbi.1999.3091

[59] Cuff, J. A. & Barton, G. J. Application of multiple sequence alignment profiles to improve protein secondary structure prediction. *Proteins* **40**, 502–511 (2000). 10861942 10.1002/1097-0134(20000815)40:3<502::aid-prot170>3.0.co;2-q

[60] Erdős, G., Pajkos, M. & Dosztányi, Z. IUPred3: prediction of protein disorder enhanced with unambiguous experimental annotation and visualization of evolutionary conservation. *Nucleic Acids Res.* **49**, W297–W303 (2021). 34048569 10.1093/nar/gkab408 PMC8262696

[61] Fleming, J. et al. AlphaFold Protein Structure Database and 3D-Beacons: New Data and Capabilities. *J. Mol. Biol.* **437**, 168967 (2025). 40133787 10.1016/j.jmb.2025.168967

[62] Vaisey, G. & MacKinnon, R. Lipid composition and mechanical force underlie multi-modal regulation of Piezo1 gating. *Sci Adv* **12**, eaed7115 (2026). 10.1126/sciadv.aed7115 PMC13232553 42234740

[63] Cox, C. D. et al. Removal of the mechanoprotective influence of the cytoskeleton reveals PIEZO1 is gated by bilayer tension. *Nat. Commun.* **7**, 10366 (2016). 26785635 10.1038/ncomms10366 PMC4735864

[64] Verkest, C. et al. Intrinsically disordered intracellular domains control key features of the mechanically-gated ion channel PIEZO2. *Nat. Commun.* **13**, 1365 (2022). 35292651 10.1038/s41467-022-28974-6 PMC8924262

[65] Syeda, R. et al. Chemical activation of the mechanotransduction channel Piezo1. *ELife* **4**, e07369 (2015). 26001275 10.7554/eLife.07369 PMC4456433

[66] Ellefsen, K. L. et al. Myosin-II mediated traction forces evoke localized Piezo1-dependent Ca 2+ flickers. *Commun. Biol.* **2**, 1–13 (2019). 31396578 10.1038/s42003-019-0514-3 PMC6685976

[67] Bertaccini, G. A. et al. Visualizing PIEZO1 localization and activity in hiPSC-derived single cells and organoids with HaloTag technology. *Nat. Commun.* **16**, 5556 (2025). 40593468 10.1038/s41467-025-59150-1 PMC12217361

[68] Szczot, M. et al. Cell-type-specific splicing of piezo2 regulates mechanotransduction. *Cell Rep.* **21**, 2760–2771 (2017). 29212024 10.1016/j.celrep.2017.11.035 PMC5741189

[69] Edelstein, A. D. et al. Advanced methods of microscope control using μManager software. *J. Biol. Methods* **1**, 1 (2014). 10.14440/jbm.2014.36 PMC4297649 25606571

[70] Gavara, N. A beginner’s guide to atomic force microscopy probing for cell mechanics. *Microsc Res. Tech.* **80**, 75–84 (2017). 27676584 10.1002/jemt.22776 PMC5217064

[71] Roettger, L., Zeitzschel, N., Gorzelanny, C., Verkest, C. & Lechner, S.G. State-dependent binding of the wedge domain controls inactivation of the mechanosensitive ion channel PIEZO.* Zenodo*10.5281/zenodo.21363175 (2026).10.1038/s41467-026-76927-042649207

