# Supplementary material for: State-dependent binding of the wedge domain controls inactivation of the mechanosensitive ion channel PIEZO1
Source: Nat Commun. 2026 Aug 26;17:9057. doi: 10.1038/s41467-026-76927-0 (PMC13518315; doi:10.1038/s41467-026-76927-0)
Supplement: Supplementary file 1 [file 41467_2026_76927_MOESM1_ESM.pdf]

Supplementary Information

Title: State-dependent binding of the wedge domain controls inactivation of the mechanosensitive ion channel PIEZO1

Authors: Lucas Roettger[1], Nadja Zeitzschel[1], Christian Gorzelanny[2], Clement Verkest[1,*] & Stefan G. Lechner[1,*]

Affiliations:

[1]Department of Anesthesiology, University Medical Center Hamburg-Eppendorf, Germany

[2]Department of Dermatology and Venereology, University Medical Center Hamburg-Eppendorf, Germany.

*Corresponding authors: c.verkest@uke.de and s.lechner@uke.de

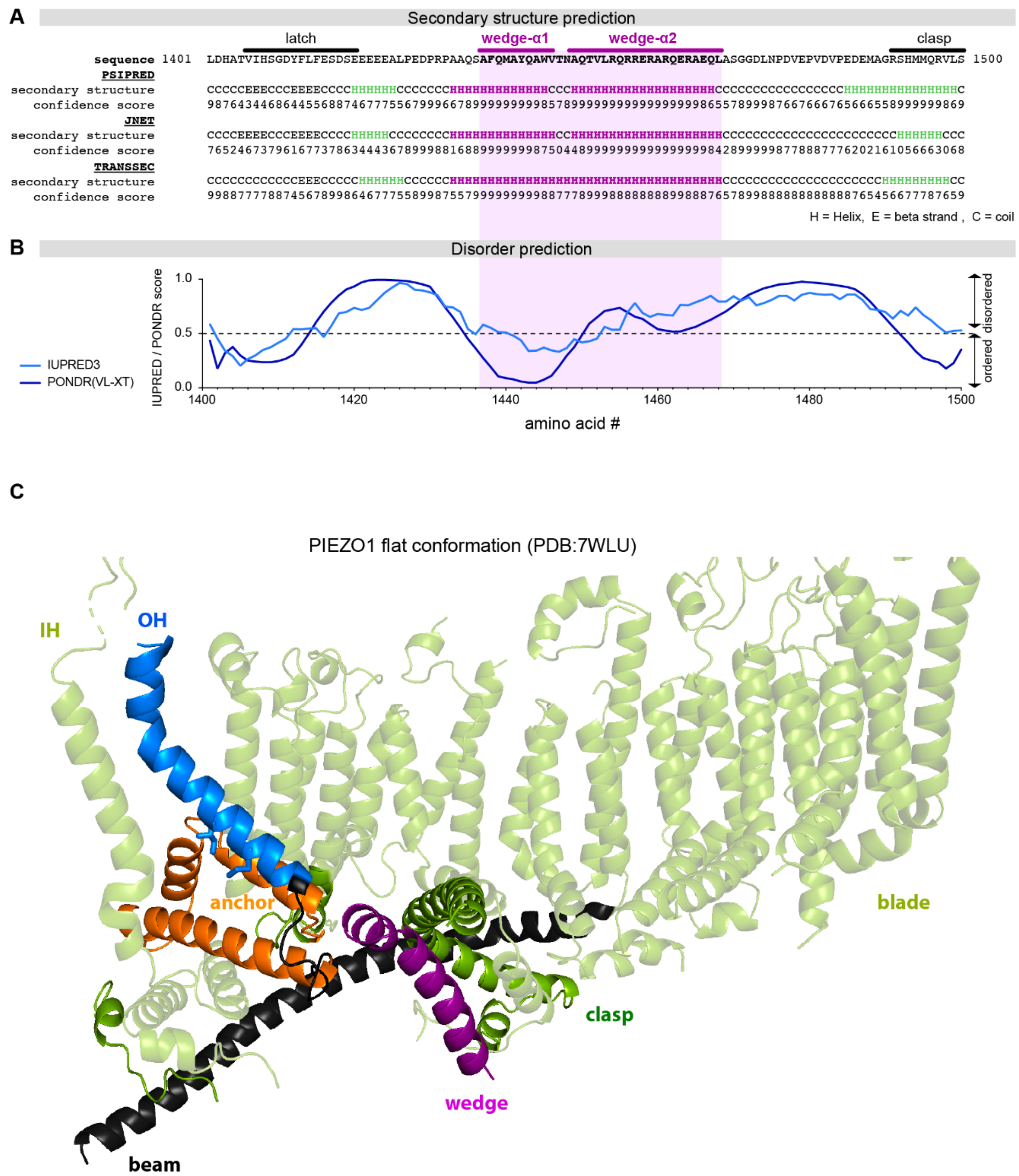

Supplementary Figure 1 | In-silico prediction of the secondary structure of the wedge and adjacent linkers

(A) The indicated amino acid sequence (top) including the wedge and the adjacent regions was submitted to the PROTEUS structure predictino server 2.0, which implements PSIPRED, JNET and the locally developed TRANSSEC algorithm for secondary structure prediction. Predicted secondary structures are indicated by H(helix), E (beta strand) and C (coil) and PROTEUS confidence scores (1-10, with 10 indicating highest confidence). Note, all three algorithms predict a helical secondary strucutre for the wedge domain.

(B) The same amino acid sequence shown in (A) was analyzed for the presence of intrinsically disordered regions using the IUPRED3 (bright blue) and PONDR(VL-XT; dark blue) algorithms. Note, the wedge, specifically the a1-helix, has a very low disorder score, whereas the adjacent linkers appear to be highly diordered.

(C) Crystal structure visualization of PIEZO1 flat conformation (PDB: 7WLU) from a single protomer, with the outer helix (OH, blue), the anchor (orange), the beam (black), the clasp (dark green) and the wedge (purple) highlighted.

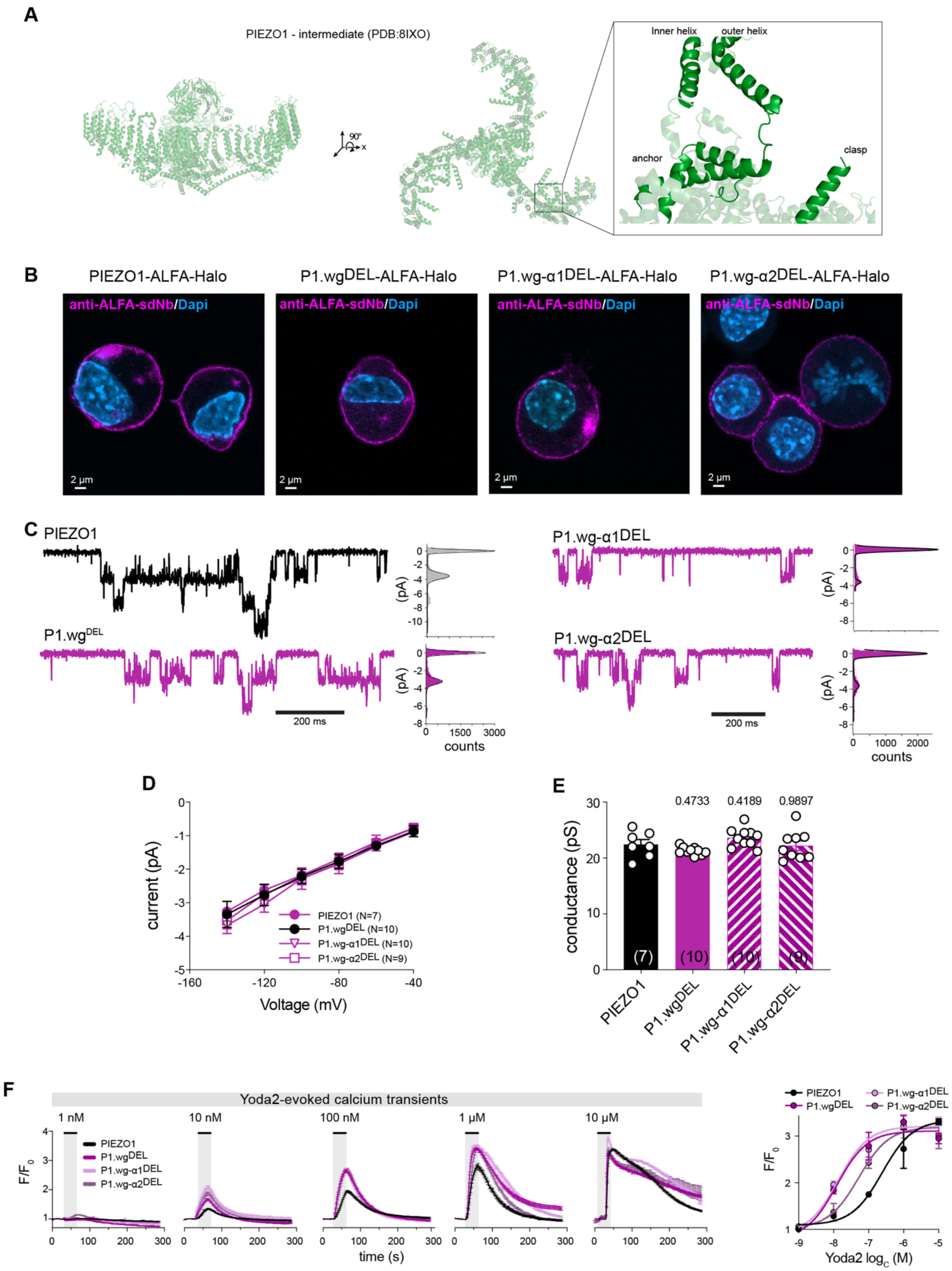

Supplementary Figure 2 | Characterization of PIEZO1 wedge deletion mutants

(A) Side (left) and top (middle) view of the intermediate open structure of the PIEZO1-S2472E mutant. Close-up view (right) shows the absence of the wedge docking to anchor-OH linker.

(B) confocal images of unpermeabilzed N2a cells expressing the the indicated PIEZO1 variants showing and labelled with an anti-ALFA nanobody recognizing the extracellulal AFLA tag, showing prominent plasma membrane expression.

(C) Example traces of stretch-evoked single channel openings of PIEZO1 (top left) and Wedge^{DEL} (bottom left), corresponding all-points histogram (right), with Gaussian fits and indicated peak average of PIEZO1and Wedge^{DEL}.

(D) Current/voltage relationship of single-channel amplitude of PIEZO1 and P1.wgDEL fitted with a linear regression. Symbols represent means ± s.e.m. and N-numbers of individual cells (biological replicates) are provided in brackets.

(E) Comparison of the mean ± s.e.m. single channel conductance, deduced from the data shown in (D), using Student's unpaired t-test with Welch's correction. Exact P-values are provided above bars, N numbers are indicated inside the bars and black circles show values from individual cells.

(F) Average time-courses of Yoda2-evoked calcium transients (left) measured as F/F0 (= GCamp8 fluorescence intensity at time t divided by the average GCamp8 intensity during the first ten frames) and dose-response curves of Yoda2 for the indicated channel mutants. Response magnitudes were averaged for all cells in the field of view on a coverslip and each Yoda2 concentration was tested on at least three coverslips. Exact numbers of tested coverslips was: PIEZO1: 1 nm Yoda2, N = 23 coverslips, 10 nM Yoda2, N=22, 100 nM Yoda2, N = 23; 1 µM Yoda2, N = 4; 10 µM Yoda2, N = 23; P1.wg^{DEL}: 1 nm Yoda2, N = 4 coverslips, 10 nM Yoda2, N=4, 100 nM Yoda2, N = 4; 1 µM Yoda2, N = 4; 10 µM Yoda2, N = 4; P1.wg-α1^{DEL}: 1 nm Yoda2, N = 4 coverslips, 10 nM Yoda2, N=5, 100 nM Yoda2, N = 4; 1 µM Yoda2, N = 4; 10 µM Yoda2, N = 5; P1.wg-α2^{DEL}: 1 nm Yoda2, N = 6 coverslips, 10 nM Yoda2, N=3, 100 nM Yoda2, N = 8; 1 µM Yoda2, N = 4; 10 µM Yoda2, N = 4.

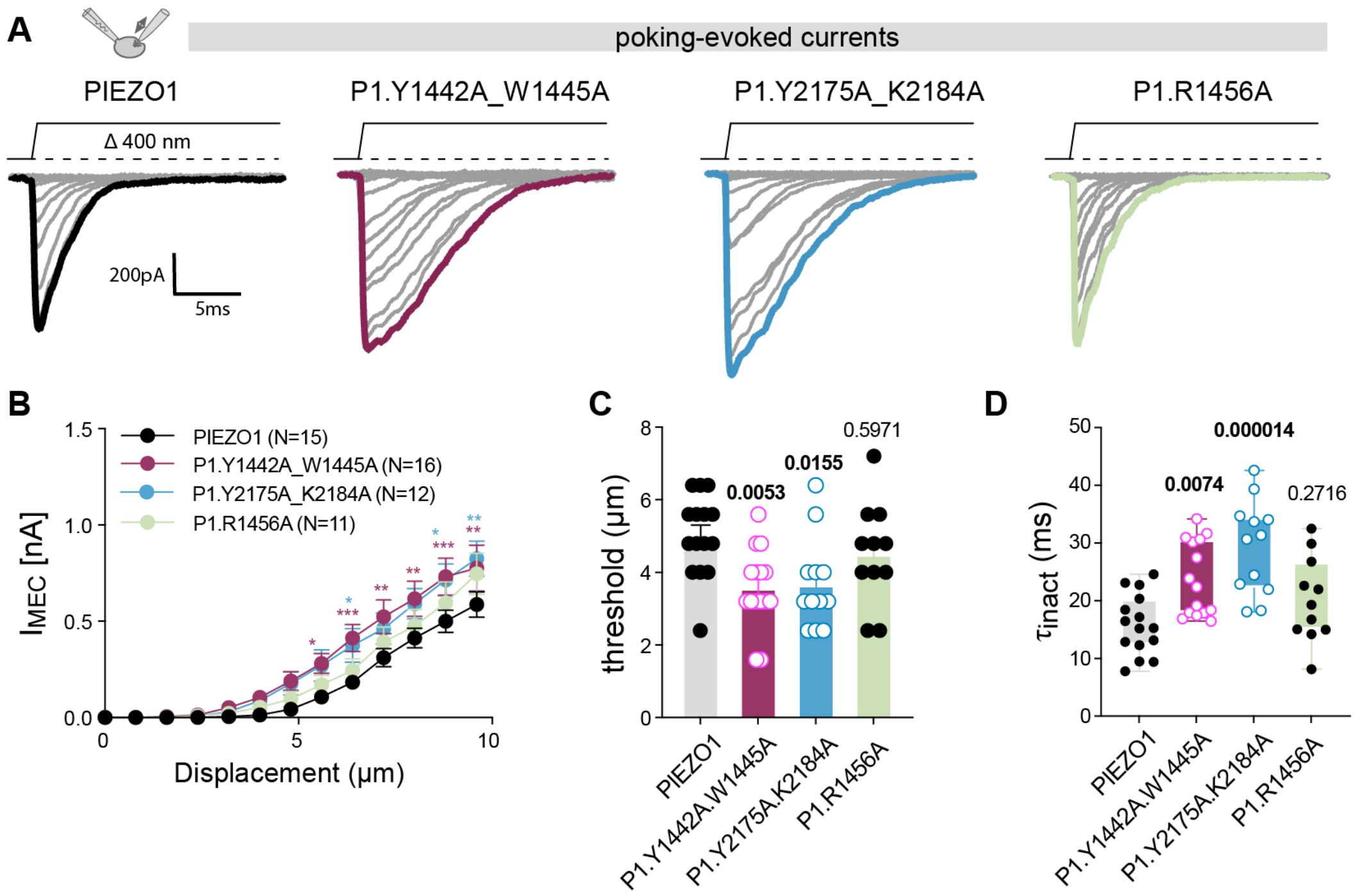

Supplementary Figure 3 | Characterization of PIEZO1 and wedge point-mutants with the poking assay

(A) Example traces of whole-cell poking-evoked currents of PIEZO1, P1.Y1442A_W1445A, P1.Y2175A_K2184A and P1.R1456A .

(B) Displacement-response curves of PIEZO1 and associated wedge point-mutants. Comparison with Dunnett's multiple comparison test. Symbols represent means ± s.e.m. and N-numbers in brackets represent number of cells recorded.

(C) Comparison of mean ± s.e.m. mechanical activation thresholds of poking-evoked currents using One-way ANOVA test with Dunnett's multiple comparison test. Exact P-values are provided above bars, N numbers are the same as in **(B)** and circles show values from individual cells.

(D) Comparison of inactivation time constants of poking-evoked currents, determined by exponential decay fits, using One-way ANOVA test with Dunnett's multiple comparison test. Exact P-values are provided above bars, N numbers are the same as in **(B)** and circles show values from individual cells.

A

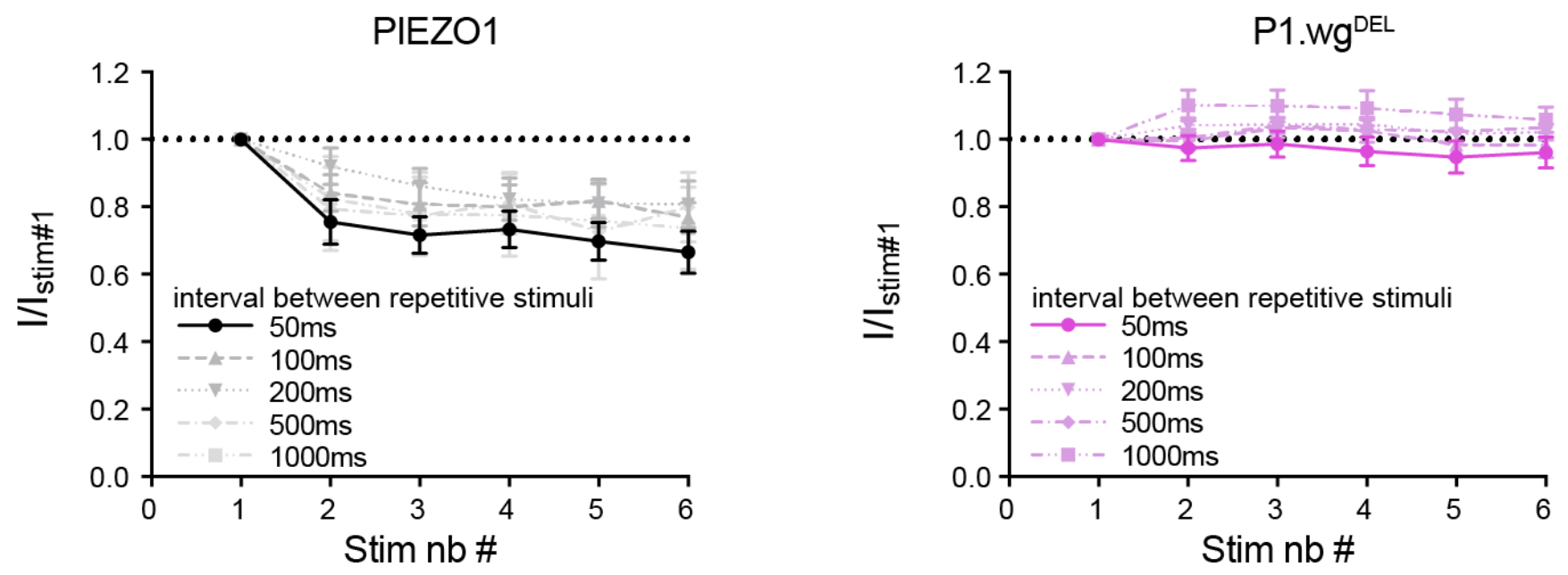

Supplementary Figure 4 | Recovery from inactivation of PIEZO1 and P1.wg^{DEL}

(A) Example traces of PIEZO1 (top left) and P1.wg^{DEL}-mediated (bottom, left) currents elicited by repetitive mechanical stimulation. Magnitude of responses evoked by repeated mechanical stimulation (200 ms, -60 mmHg) at the indcated time intervals and normalized to the amplitude of the response evoked by the first stimulus in cells expressing PIEZO1 (left) and P1.wg^{DEL} (right). Symbols represent means ± s.e.m. from N individual cells, with N being: PIEZO1: 50 ms, N=9; 100 ms, N = 7; 200 ms, N = 8; 500 ms, N = 6; 1000 ms, N = 8. P1.wg.DEL: 50 ms, N = 13; 100 ms, N = 13; 200 ms, N = 13; 500 ms, N = 14; 1000 ms, N = 14.

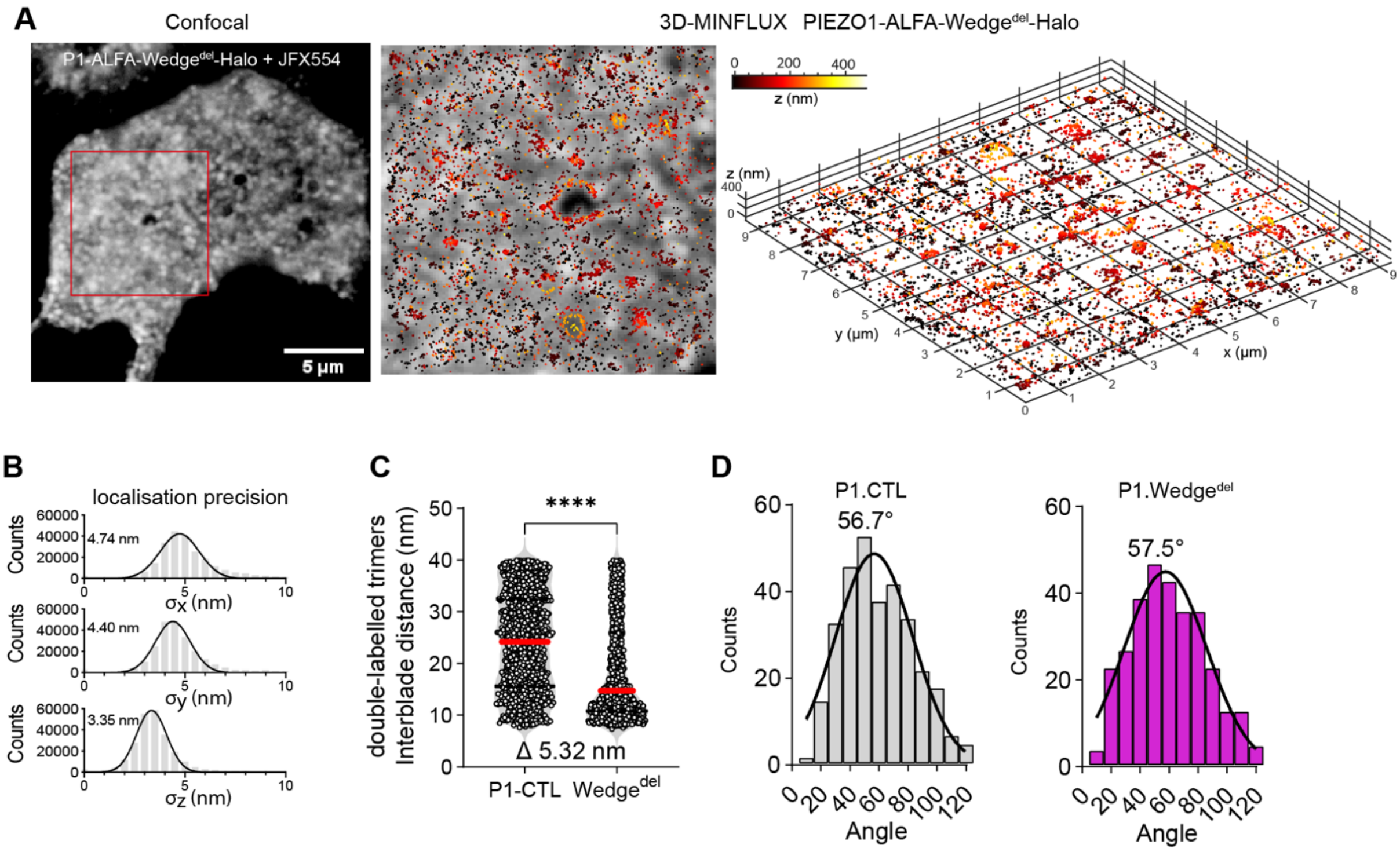

Supplementary Figure 5 | 3D-MINFLUX characterization of PIEZO1 and P1-wgDEL

(A) Confocal image of P1.wg^{DEL} expressing N2a.P1KO cell (left), close-up view of region indicating by red square with superimposed MINFLUX raw data (middle), and perspective view of the MINFLUX data only from the same region.

(B) Histograms of standard deviations of trace locations in x (top), y (middle) and z (bottom) direction in nanometres and Gaussian fits. Respective µ-values of the Gaussian fits are indicated next to the peaks and the total number of Minflux traces from which the histograms were calculated was 18698.

(C) Comparision of interblade distances measured in double-labelled PIEZO1 and P1.wg^{DEL} trimers using Mann-Whitney test (****, P< 0.0001). Medians are indicated as red lines, quartiles as dotted black lines and delta of means is indicated at the bottom. Number of double labelled dimers: P1-CTL = 1101, Wedge^{del} = 822.

(D) Histograms of the interblade angles (see Fig. 4B right panel for explanation) of the triple labelled PIEZO1 (left) and P1.wg^{DEL} (right) trimers with Gaussian fit and means indicated above peak. N-number equal 3 times the N-numbers in Fig. 3C (P1-CTL, N=315; P1.Wedge^{del}, N = 309), because each trimer has three vertices.

Supplementary Table 1. List of primers used for generation of PIEZO1 mutants

Plasmid Name	Primer	Sequence
P1_ALFA_Halo	Vector_FWD	TAAAGGCGCTCCTGCCCTGGCAACCA
	Vector_REV	GAGGTTCCATGGTGGCGCGGCC
	Fragment_FWD	CCGCGCCACCATGGAACCTCACGTGCTCG
	Fragment_REV	CCAGGGCAGGAGCGCCTTTAAGTCCCACAG
P1_ALFA_Halo_Y1442A	FWD	CCTTCCAGATGGCAGCCCAGGCATGGGTAACCAATGC
	REV	GCATTGGTTACCCATGCCTGGGCTGCCATCTGGAAGG
P1_ALFA_Halo_W1445A	FWD	CCAGATGGCATACCAGGCAGCGGTAACCAATGC
	REV	GCATTGGTTACCGCTGCCTGGTATGCCATCTGG
P1_ALFA_Halo_R1456A	FWD	GACAGTGCTGAGGCAGGCGCGGGAGCGGGCACGG
	REV	CCGTGCCCGCTCCCGCGCCTGCCTCAGCACTGTC
P1_ALFA_Halo_Y2175A	FWD	GACAGAGAAGAAAGCCCCCCAGCCCAAGG
	REV	CCTTGGGCTGGGGGGCTTTCTTCTCTGTC
P1_ALFA_Halo_K2184A	FWD	GGGGCAGAAGAAGGCGAAAATTGTCAAGTATGG
	REV	CCATACTTGACAATTTTCGCCTTCTTCTGCCCC
P1_ALFA_Halo_Y1442A-W1445A	FWD	GAGTGCCTTCCAGATGGCAGCCCAGGCAGCGGTAACC
	REV	GGTTACCGCTGCCTGGGCTGCCATCTGGAAGGCACTC
P1_ALFA_Halo_wg^{DEL}	Vector_FWD	CCTGCATATCCCTGAGCTGGAG
	Vector_REV	TCAAGTCACCCTGAGCTGCAGGCCTGG
	Fragment_FWD	TGCAGCTCAGGGTGACTTGAACCCAGATGTGGAAC
	Fragment_REV	CCAGCTCAGGGATATGCAGGCG
P1_ALFA_Halo_wgα2^{DEL}	Vector_FWD	CCTGCATATCCCTGAGCTGGAG
	Vector_REV	TCAAGTCACCATTGGTTACCCATGCCTGGTATG
	Fragment_FWD	GGTAACCAATGGTGACTTGAACCCAGATGTGGAAC
	Fragment_REV	CCAGCTCAGGGATATGCAGGCG
P1_ALFA_Halo_wgα1^{DEL}	Vector_FWD	CCTGCATATCCCTGAGCTGGAG
	Vector_REV	CTGTCTGGGCCTGAGCTGCAGGCCTGG
	Fragment_FWD	TGCAGCTCAGGCCCAGACAGTGCTGAGGCA
	Fragment_REV	CCAGCTCAGGGATATGCAGGCG
P1_ALFA_Halo_Y2175A_K2184A	FWD	GACAGAGAAGAAAGCCCCCCAGCCCAAGG
	REV	CCTTGGGCTGGGGGGCTTTCTTCTCTGTC

Supplementary Table 2 | 3D-MINFLUX scan sequence parameters

Iteration	Modality	Pattern diameter (nm)	Photon limit	Back-ground limit	Pattern dwell time (ms)	Pattern repeat	Sticki ness	CFR limit	Laser power factor
1	Hexagonal (confocal)	288	160	15000	1	1	-	none	1
	zline	288	400	15000	1	1	2	none	1
2	square	288	100	10000	1	5	2	none	1
	zline2	288	50	10000	1	5	2	none	1
3	square	151	67	10000	1	5	2	0.9	2
	zline2	151	33	10000	1	5	2	none	2
4	square	76	67	10000	1	5	2	0.8	4
	zline2	76	33	10000	1	5	2	none	4
5	square	40	100	10000	1	5	2	none	6
	zline2	40	50	10000	1	5	2	none	6
