# Supplementary material for: State-dependent binding of the wedge domain controls inactivation of the mechanosensitive ion channel PIEZO1
Source: Nat Commun. 2026 Aug 26;17:9057. doi: 10.1038/s41467-026-76927-0 (PMC13518315; doi:10.1038/s41467-026-76927-0)
Supplement: Supplementary file 3 [file 41467_2026_76927_MOESM3_ESM.pdf]

nature portfolio

Peer Review File

State-dependent binding of the wedge domain controls inactivation of the mechanosensitive ion channel PIEZO1

Corresponding Author: Professor Stefan Lechner

This file contains all reviewer reports in order by version, followed by all author rebuttals in order by version.

Attachments originally included by the reviewers as part of their assessment can be found at the end of this file.

Version 0:

Reviewer comments:

Reviewer #1

(Remarks to the Author)
This interesting manuscript by Roettger et al. focuses on understanding the molecular basis of inactivation in PIEZO1 channels. The molecular/structural basis of inactivation in PIEZO1/2 is a really important open research question. It relates to both disease-causing mutations and the regulation of endogenous PIEZO1/2 inactivation by native binding partners, as most primary cells exhibit slower inactivation. Here the authors identify an intracellular wedge domain that influences inactivation. The data is beautiful, it's well rationalised, it's well described and the narrative places it perfectly into the broader landscape of the PIEZO literature. I have a few questions and comments below that I think should be addressed/discussed prior to publication.

1 – Small influence on poking induced inact?
The cell-attached recordings in figure 3 particularly are really beautiful and very convincing. This makes it difficult for me to rationalise the magnitude of the effect on Tau inact in whole-cell poking. MDFIC binding or disease mutants have much bigger effects on inact than this. Surely at the very least it should be discussed that this effect on tau is very small compared to the effects seen in Figure 3. This is also important because the abstract and text suggest the loss of the wedge domain "abolishes inactivation" and clearly there is still inact in whole-cell. If the authors have alternate molecular interpretations of the "adaptive" kinetics still present in the whole-cell configuration I for one would be super interested to hear them. If not, the language should be modified and the difference in magnitude between cell-attached and whole-cell described.

2- Conservation in PIEZO2
While reading an obvious question is whether this domain is also important in PIEZO2 inactivation? Clearly the auxiliary proteins MDFI, MDFIC and MDFIC2 all bind to this site and influence PIEZO2 inactivation [Koster et al., 2024 PNAS, Habib et al., 2025 PNAS, Zhou et al., 2025 BioRxiv]. An explanation that spans PIEZOs would be even more convincing to this reviewer. If the authors have ongoing studies targeting PIEZO2 they can at least leave the question there in the discussion as to whether this is a mechanism also utilised by PIEZO2.

Line 56 / 86 - This site docks all the MyoD-family inhibitors not just MDFIC. MDFI and MDFIC2 [Koster et al., 2024 PNAS, Shan et al., 2024 eLife, Habib et al., 2025 PNAS, Zhou et al., 2025 BioRxiv].
Line 309 – Related to the above point these regulators give exceptionally different degrees of slowing see Koster et al., 2024 PNAS as an example of this (shows CADM1/CDH2/MDFI).
Line 199 – "Considering that PIEZO1 only shows little spontaneous activity in the absence of mechanical stimulation" – what is the rationale for this comment? It seems superfluous. Later discussion around line 321 mentions correctly that cell generated forces clearly influence PIEZO1 activity without exogenous mechanical load, in my view best shown in Ellefsen et al., 2019 Comms Biol.
Line 412 – realistically I think in Figure 2B that SERCA2A, cadherin and PIPs should be referred to as putative binding partners until they are unequivocally proven and replicated.

(Remarks on code availability)

Reviewer #2

(Remarks to the Author)
Piezo channels are involved in many physiological processes, and Piezo inactivation is a critical parameter for the channel's function. Here, the authors claim that the wedge domain is a critical determinant of Piezo1 inactivation, acting via the ball-and-chain mechanism, whereby the wedge physically occludes the pore, similar to some voltage-gated ion channels. However, the data do not support either conclusion. At most, the authors show that in the absence of the wedge domain, inactivation is mildly altered, but not eliminated (as would be expected from a 'critical' domain. Te MINIFLUX data suggest that the wedge domain changes the distribution of Piezo1 conformations toward more curved ones, but the ball-and-chain mechanism is not explicitly tested, leaving this conclusion entirely speculative. This study that reveals a new (and modest) role for the wedge domain in Piezo1 inactivation, but it is rather incremental and does not establish a novel mechanistic paradigm, as the authors claim.

Major

The deletion of the wedge domain apparently eliminates inactivation in the stretch assay (1C), but in the poking assay the effect is minimal (1F). For comparison, many mutations have been described with far more pronounced effects or with complete elimination of inactivation. The rather mild effect of wedge deletion on Piezo1 inactivation (1F, G) at the very onset of the study argues against its role as a 'critical determinant' of this process.

While stretching is useful for measuring the Hg50, it is far less precise when it comes to measuring the timing of inactivation, and Piezo1 usually responds to the stretch assay with much slower inactivation than to poking. What the authors observe here could be an epi-phenomenon specific to the stretch assay, which is very sensitive to the geometry of the pipette and has many confounds, such as increased basal membrane tension in the patch, (which can alter inactivation), seal creep, etc. Indeed, the authors couldn't extract the tau from this assay and instead used the "peak/sustained" ratio. At the same time, the poking assay, which allows precise estimation of inactivation, shows that the effect size of wedge deletion on inactivation is very mild, increasing the tau only twofold, but inactivation is still visibly there and pronounced.
Fig. 1C vs D. Quantification of peak/sustained in D (~2) doesn't seem to match the exemplary currents in C, where the ratio is closer to 1.
Fig. 2. The authors convincingly show that the mutants slow inactivation. However, the conclusion that "in the flat (open) state – the wedge binds to the anchor-OH-linker…, thereby promoting rapid inactivation" is speculative (and so is the title of Fig 2). The authors refer to published structures, but there is nothing in the presented data that supports such a nuanced mechanistic conclusion. It would also be critical to test the importance of these mutants in the more sensitive poking assay.

Fig. 2E. What the authors have really shown is that the wedge domain contains residues that influence the sensitivity of the channel to membrane stretch (decrease Hg50). This, rather than inactivation, could be its primary role.

Fig. 4. The data do not support the conclusion that the increased interblade distance necessarily implies that a higher proportion of the channels is in the inactivated conformation (as opposed to 'closed', deactivated, or some other), or that it suggests the "ball-and-chain mechanism". The authors do not present any data that the wedge acts as a ball that plugs the pore, this mechanism is not tested and the conclusion is speculative. The decreased proportion of 'flat' channels is also consistent with the mild overall effect of the wedge on inactivation.

Minor

Fig. S2B It is not immediately obvious how these images convey the idea that the channels are on the plasma membrane. Please explain/elaborate.

Line 111. The text implies that all three mutants were characterized by single-channel analysis, but the figure shows only one. Please correct.

(Remarks on code availability)

Reviewer #3

(Remarks to the Author)

(Remarks on code availability)

Reviewer #4

(Remarks to the Author)
I co-reviewed this manuscript with one of the reviewers who provided the listed reports. This is part of the Nature

Communications initiative to facilitate training in peer review and to provide appropriate recognition for Early Career Researchers who co-review manuscripts.

(Remarks on code availability)
Did not review code.

Version 1:

Reviewer comments:

Reviewer #1

(Remarks to the Author)
"even in the complete absence of inactivation, poking-evoked currents will still substantially decay during sustained indentation simply because the force acting on the cell declines resulting in deactivation of a considerable proportion of channels. In this context it is also noteworthy that the auxiliary PIEZO1 subunits MDFIC and MDFIC2, which are mentioned further below by this reviewer, also impair deactivation (i.e. closure of the channel upon stimulus removal)"
The new data added is very nicely rationalised and well presented. I disagree on the extension of this to the effect of MDFI/C2 on deact as this effect is strongly voltage dependent (and by definition must be an inact effect and not deact). But the point is well taken and the AFM data very strongly supports the authors interpretation.

"Also, we could not find any mention of CDH2 (aka N-Cadherin) in Koster et al."
Figure 2 shows data for CDH2 and supports the idea that cadherins have little to no effect on PIEZO1 inactivation hence my suggestion to add – "putative binding partners".

Overall all of the changes are well described and well thought out. I strongly support publication.

(Remarks on code availability)
N/A

Reviewer #2

(Remarks to the Author)
My main criticism of this manuscript is that deletion of the wedge domain has only a mild effect on inactivation in the poking assay, making it unlikely to be the critical determinant.
I agree with the authors that the poking assay has its limits and confounds, such as deactivation. Yet the strikingly mild effect observed in the poking assay, compared to the other mutations and conditions that the authors enumerated in the rebuttal, suggests that the critical determinant may lie elsewhere in the channel. The authors argue that the apparent discrepancy between the assays is because of 'deactivation' during poking. While this is possible, there is also a tangible possibility that the results seen in the stretch assay reflect patch mechanics inherent to the method itself. Greater agreement between the assays would provide more confidence that this domain is indeed critical.
I am happy that the authors have taken this into consideration and toned down their language in the abstract (abolishes -> diminishes), and I'd encourage them to make similar adjustments throughout the manuscript (e.g. the Figure 1 legend still uses "abolishes").
The addition of a thorough discussion addressing the discrepancy between the two assays is also commendable. For balance, I would suggest including a few sentences on the limitations and confounds of the stretch assay (e.g., seal creep, increased basal membrane tension, and tip geometry).
Overall, this manuscript is overly definitive in assigning the ball-and-chain-like role to the wedge domain and in claiming its critical role in inactivation. That said, the actual data are very solid, the topic is highly important, and the study would be of great interest to the community.

(Remarks on code availability)

Reviewer #4

(Remarks to the Author)
The article makes interesting contributions to the broader understanding of PIEZO1. The authors use electrophysiology, and MINFLUX nanoscopy measurements to understand inactivation regulation of PIEZO1.

Upon preliminary inspection, the experimental design of the manuscript appears robust. However, closer inspection reveals that certain elements in the experimental design and the narrative require closer scrutiny.

The reviewer would recommend the article for publication provided the following concerns are addressed, either through explanations in the manuscript, or through additional control experiments. The comments have been annotated by line number.

Line 852 – 854: A few concerns with this sentence, and broadly, the MINFLUX methods section.

1. Have a table showing the acquisition parameters for each iteration. It is slowly becoming standard, and good practice in MINFLUX literature. Look up the kinesin tracking paper from Jonas Ries's group, or Stefan Hell's group, to see how such a table should be formatted. Even if it is the manufacturer's default sequence file, the information relevant to that must be included.

2. How confident are the authors of the study that they used the manufacturer's default sequence file, with no alterations? In the reviewer's experience, (and from anecdotes from other users of the MINFLUX), the default sequence file of the manufacturer can be very difficult in providing 1 nm – 5 nm localization precisions, unless significant sequence file edits are done. If requirement 1 above is fulfilled, the reviewer would have greater confidence in the robustness of the MINFLUX acquisition method. The Methods section needs to illustrate how it was done, and the credibility of the measurement must be further bolstered.

3. Was the localization of <5 nm achieved with stationary fluorophores? i.e.: were stationary controls done to distinguish between fluorophore movement and instrument noise? The reviewer would recommend this as a confirmatory control study. Just put down ATTO655 on a coverslip, with no cells, no movement of the fluorophores, and record 3D images of the fluorophores. Use the same methodology that was previously used to calculate localization precision. See if 1 nm can still be achieved.

4. How was the localization precision calculated? Or was it reported directly from manufacturer-provided widgets such as ParaFLUX or FilterFLUX? The methodology and equations used, must be specified.

(Remarks on code availability)

Open Access This Peer Review File is licensed under a Creative Commons Attribution 4.0 International License, which permits use, sharing, adaptation, distribution and reproduction in any medium or format, as long as you give appropriate credit to the original author(s) and the source, provide a link to the Creative Commons license, and indicate if changes were made.
In cases where reviewers are anonymous, credit should be given to 'Anonymous Referee' and the source.
The images or other third party material in this Peer Review File are included in the article's Creative Commons license, unless indicated otherwise in a credit line to the material. If material is not included in the article's Creative Commons license and your intended use is not permitted by statutory regulation or exceeds the permitted use, you will need to obtain permission directly from the copyright holder.
To view a copy of this license, visit https://creativecommons.org/licenses/by/4.0/

REVIEWER COMMENTS

Reviewer #1 (Remarks to the Author):

This interesting manuscript by Roettger et al. focuses on understanding the molecular basis of inactivation in PIEZO1 channels. The molecular/structural basis of inactivation in PIEZO1/2 is a really important open research question. It relates to both disease-causing mutations and the regulation of endogenous PIEZO1/2 inactivation by native binding partners, as most primary cells exhibit slower inactivation. Here the authors identify an intracellular wedge domain that influences inactivation. The data is beautiful, it's well rationalised, it's well described and the narrative places it perfectly into the broader landscape of the PIEZO literature. I have a few questions and comments below that I think should be addressed/discussed prior to publication.

1 – Small influence on poking induced inact? The cell-attached recordings in figure 3 particularly are really beautiful and very convincing. This makes it difficult for me to rationalise the magnitude of the effect on Tau inact in whole-cell poking. MDFIC binding or disease mutants have much bigger effects on inact than this. Surely at the very least it should be discussed that this effect on tau is very small compared to the effects seen in Figure 3. This is also important because the abstract and text suggest the loss of the wedge domain "abolishes inactivation" and clearly there is still inact in whole-cell. If the authors have alternate molecular interpretations of the "adaptive" kinetics still present in the whole-cell configuration I for one would be super interested to hear them. If not, the language should be modified and the difference in magnitude between cell-attached and whole-cell described.
This is an excellent point and we agree that this point requires further attention. It is important to note that in the poking assay, current decay cannot be unambiguously attributed to inactivation, defined as transition from the active to an inactive state during continued stimulation. It may also reflect deactivation, i.e. transition from the active to the closed/resting state, because the effective stimulus declines over time due to relaxation of the indented cell, such that the forces may drop below the activation threshold. This limitation had previously been proposed by Lewis and Grandl in a comprehensive comparison of the stretch- and poke assays (PMID: 34120715) and was subsequently demonstrated by Young et al. (PMID: 36795747), who observed a rapid decay of indentation forces during mechanical indentation of HEK293 cell using atomic force microscopy (AFM).
Inspired by the reviewer's comment, we have thus measured the temporal dynamics of indentation forces during cell indentation with AFM. The new data clearly shows that N2a cells (as previously shown for HEK293 cells) adapt to mechanical indentation, such that the indentation forces rapidly decline to levels below 40% of the initial peak force (new Fig. 1I). Thus, even in the complete absence of inactivation, poking-evoked currents will still substantially decay during sustained indentation simply because the force acting on the cell declines resulting in **de**activation of a considerable proportion of channels. In this context it is also noteworthy that the auxiliary PIEZO1 subunits MDFIC and MDFIC2, which are mentioned further below by this reviewer, also impair deactivation (i.e. closure of the channel upon stimulus removal), which may explain why these two proteins also have larger effects on the current decay during stimulation (PMID: 37590348, PMID: 41201821, PMID: 41955114).
We hope the new data and the detailed discussion of this point (both highlighted in yellow in the revised manuscript) resolve this issue.

2- Conservation in PIEZO2   While reading an obvious question is whether this domain is also important in PIEZO2 inactivation? Clearly the auxiliary proteins MDFI, MDFIC and MDFIC2 all bind to this site and influence PIEZO2 inactivation [Koster et al., 2024 PNAS, Habib et al., 2025 PNAS, Zhou et al., 2025 BioRxiv]. An explanation that spans PIEZOs would be even more convincing to this reviewer. If the authors have ongoing studies targeting PIEZO2 they can at least leave the question there in the discussion as to whether this is a mechanism also utilised by PIEZO2.
We agree that understanding the role of the wedge in PIEZO2 function would also be interesting. We did not investigate that for several reasons. First, it had previously been shown that exon 35, which encodes the wedge domain of PIEZO2, is alternatively spliced and PIEZO2 splice variants lacking this exon (together with others) indeed exhibit slower inactivation (Szczot et al Cell Rep 2017) although a recent preprint reports conflicting results (Sindoni et al. bioRxiv 2026). Second, the flat structure of PIEZO2 has not been resolved yet, so it is unclear if and where the wedge binds to PIEZO2 in the flat state, such that meaningful mechanistic conclusions about a possible role of the wedge in PIEZO2 inactivation would be impossible. We have emphasized that splicing of exon 35 also affects inactivation of PIEZO2 in the revised manuscript. We hope this explanation satisfies the reviewer and clarifies why we did not dig deeper into PIEZO2.

Line 56 / 86 - This site docks all the MyoD-family inhibitors not just MDFIC. MDFI and MDFIC2 [Koster et al., 2024 PNAS, Shan et al., 2024 eLife, Habib et al., 2025 PNAS, Zhou et al., 2025 BioRxiv].
We added MDFIC2 together with the relevant citations to the text and to Fig 1A (MDFI was already listed in the original version - line 57) as suggested by the reviewer.

Line 309 – Related to the above point these regulators give exceptionally different degrees of slowing see Koster et al., 2024 PNAS as an example of this (shows CADM1/CDH2/MDFI).
We appreciate that the reviewer points out that CADM1 has a smaller effect on inactivation than MDFI but we are not quite sure how this information is relevant for our statement in line 309, where we speculated that a possible mode-of-action of the wedge is that it directly competes with the afore-mentioned modulators or indirectly blocks access to their binding sites such that they can exert stronger effects (i.e. slow inactivation) in the absence of the wedge. Also, we could not find any mention of CDH2 (aka N-Cadherin) in Koster et al.

Line 199 – "Considering that PIEZO1 only shows little spontaneous activity in the absence of mechanical stimulation" – what is the rationale for this comment? It seems superfluous.
The purpose of this sentence was to emphasize that background PIEZO1 activity in the absence of external mechanical stimuli (possibly due to cell-generated forces) is extremely low and therefore the large proportion of channels that are captured in the flat conformation by MINFLUX, unlikely represent an open state but rather a flat inactive state. We have modified the sentence accordingly and hope that this resolves this issue.

Later discussion around line 321 mentions correctly that cell generated forces clearly influence PIEZO1 activity without exogenous mechanical load, in my view best shown in Ellefsen et al., 2019 Comms Biol.
We thank the reviewer for pointing out the missing reference and have added a reference to the Ellefsen paper to resolve this issue.

Line 412 – realistically I think in Figure 2B that SERCA2A, cadherin and PIPs should be referred to as putative binding partners until they are unequivocally proven and replicated.

Agreed – we have labelled them as 'putative' in Fig.2B as suggested by the reviewer.

Reviewer #2 (Remarks to the Author):

Piezo channels are involved in many physiological processes, and Piezo inactivation is a critical parameter for the channel's function. Here, the authors claim that the wedge domain is a critical determinant of Piezo1 inactivation, acting via the ball-and-chain mechanism, whereby the wedge physically occludes the pore, similar to some voltage-gated ion channels. However, the data do not support either conclusion. At most, the authors show that in the absence of the wedge domain, inactivation is mildly altered, but not eliminated (as would be expected from a 'critical' domain. Te MINIFLUX data suggest that the wedge domain changes the distribution of Piezo1 conformations toward more curved ones, but the ball-and-chain mechanism is not explicitly tested, leaving this conclusion entirely speculative. This study that reveals a new (and modest) role for the wedge domain in Piezo1 inactivation, but it is rather incremental and does not establish a novel mechanistic paradigm, as the authors claim.

We thank the reviewer for taking the time to review our paper and for providing critical feedback. Before addressing the individual major and minor points in detail, we would, however, like to clarify some of the points the reviewer has raised in his/her introductory statement.

First, we neither claimed nor insinuated that the wedge *'…physically occludes the pore'* as stated by the reviewer. In fact, we had been very careful with our wording. We made it very clear that we think that the wedge serves as an inactivation particle that *"… terminates ion flux during sustained mechanical stimulation by binding to the anchor-OH-linker"* (line 292) or *"… contributes to inactivation by binding to the anchor-OH-linker in a ball-and-chain like manner"* (line 333). Moreover, we would like to clarify that the ball-and-chain inactivation model of voltage-gated channels does NOT imply direct pore occlusion by the 'ball'. In fact recent research suggests that in voltage-gated sodium channels the 'ball' may bind adjacent to the pore and thus a novel and refined lock-and-key model has been proposed, as recently reviewed in detail by Bezanilla and colleagues (Liu et al. Nat Commun 2025, PMID: 41298396).

Second, we would like to point out that the reviewer contradicts him/herself. Above, he/she states that *"At most, the authors show that in the absence of the wedge domain, **inactivation is** mildly altered, but **not eliminated**…"* whereas in the first line of his/her major points below he/she wrote that *"…deletion of the wedge domain **apparently eliminates inactivation** in the stretch assay"*. It is very difficult to reply to and to improve the manuscript during the revision based on such diametrically opposing and contradictory statements.

Third, the reviewer states that *"…the ball-and-chain mechanism is not explicitly tested*" and therefore our conclusions are *"…entirely speculative*". We were quite puzzled when we read this comment, because the whole paper focusses on testing this mechanism. It seems like the reviewer has a different idea about how to test the ball-and-chain mechanism, but since he/she doesn't share his/her ideas with us, we can only speculate about what data he/she would like

to see to be fully convinced. We believe that three cryo-EM structures of the curved state and two cryo-EM structures of the flat state (the second one has recently been resolved by the MacKinnon lab), from three independent labs that clearly show state-dependent binding of the wedge domain together with our data, which shows that inactivation is compromised in the absence of the wedge, are very compelling evidence.

I guess that part of the confusion maybe came from our use of the term 'ball-and-chain' – note we actually used ball-and-chain-LIKE. While we chose this terminology/analogy to emphasize that the wedge is a mobile inactivation particle (i.e. ball) that is attached to the rest of the channel via flexible (i.e. chain-like) linkers, some readers – including the reviewer – may associate and imply additional mechanistic features, such as pore occlusion, with this terminology. This is understandable considering that the ball-and-chain model had been proposed almost 50 years ago and has been described in countless textbooks and review articles thereby creating fixed ideas about its implications – including direct pore block by the ball – despite recent advances in the field suggesting otherwise (see afore-mentioned review by Bezanilla and colleagues)

Major

The deletion of the wedge domain apparently eliminates inactivation in the stretch assay (1C), but in the poking assay the effect is minimal (1F). For comparison, many mutations have been described with far more pronounced effects or with complete elimination of inactivation. The rather mild effect of wedge deletion on Piezo1 inactivation (1F, G) at the very onset of the study argues against its role as a 'critical determinant' of this process.
We agree that this is an interesting point and it is a concern shared by reviewer #1. As explained in our reply to reviewer #1 (see above) it is important to acknowledge that in the poking assay, current decay cannot be unambiguously attributed to inactivation, defined as transition from the active to an inactive state during continued stimulation. It may also reflect deactivation, i.e. transition from the active to the closed/resting state, because the effective stimulus declines over time due to relaxation of the indented cell, such that the forces may drop below the activation threshold. This limitation had previously been proposed by Lewis and Grandl in a comprehensive comparison of the stretch- and poke assays (PMID: 34120715) and was subsequently demonstrated by Young et al. (PMID: 36795747), who observed a rapid decay of indentation forces during mechanical indentation of HEK293 cell using atomic force microscopy (AFM).
To address this issue, we measured the temporal dynamics of indentation forces during cell indentation with AFM. The new data (new Fig. 1I) clearly shows that N2a cells (as previously shown for HEK293 cells) adapt to mechanical indentation, such that the indentation forces rapidly decline to levels below 40% of the initial peak force. Thus, even in the complete absence of inactivation, poking-evoked currents will still substantially decay during sustained indentation simply because the force acting on the cell declines, resulting in **de**activation of a considerable proportion of channels. We have added the new data together with a detailed discussion of this issue (which also contains other possible explanations) to the revised version of the manuscript and hope that this resolves this issue.

In the paragraph above the reviewer further states that *"… many mutations have been described with far more pronounced effects or with complete elimination of inactivation"*. I am not quite sure which mutations the reviewer refers to, because unfortunately he/she did not provide any references for his/her statement. Most mutations I am aware of slow inactivation

in the poking assay by 2-5 fold (e.g. disease-causing mutations R1943Q, M2225R, R2302H, R2456H, R2488Q – a nice side-by-side comparison can be found in Romero et al. Nat Commun 2019), which is admittedly more pronounced than the effect we observed. However, to the best of our knowledge **mutations that completely eliminate inactivation in the poking assay have not been described**. The only mutation that appears to eliminate inactivation in the poking assay is the L2475/V2476/ M2493/F2494-QQQQ quadruple mutant described by Zheng et al. (elife, 2019) and the only protein interaction partners that do so are MDFIC and MDFIC2 (PMID: 37590348, PMID: 41201821, PMID: 41955114), which are mentioned by reviewer #1.

However, two important factors must be taken into account when interpreting this data. First, the quadruple mutation, MDFIC and MDFIC2 also diminish **de**activation (i.e. currents do not return to baseline after removal of the stimulus) and second, the decay of poking-evoked currents reflects a mixture of inactivation and **de**activation, as explained earlier. Thus, the reason why currents from the quadruple mutant, P1+MDFIC and P1+MDFIC2 fail to decay during sustained stimulation in the poking assay is because in addition to inactivation, deactivation is also compromised.

While stretching is useful for measuring the Hg50, it is far less precise when it comes to measuring the timing of inactivation, and Piezo1 usually responds to the stretch assay with much slower inactivation than to poking. What the authors observe here could be an epi-phenomenon specific to the stretch assay, which is very sensitive to the geometry of the pipette and has many confounds, such as increased basal membrane tension in the patch, (which can alter inactivation), seal creep, etc. Indeed, the authors couldn't extract the tau from this assay and instead used the "peak/sustained" ratio. At the same time, the poking assay, which allows precise estimation of inactivation, shows that the effect size of wedge deletion on inactivation is very mild, increasing the tau only twofold, but inactivation is still visibly there and pronounced.

Reading this comment and some of the comments below, I got the impression that the reviewer is firmly convinced that the poking assay is far better suited to measure inactivation than the stretch assay. With all due respect for the reviewer's expertise in the field, but I disagree with his/her assessment for the following reasons:

In addition to the limitations caused by stimulus adaptation, which confound the measurement of inactivation in the poking assays (**as explained in detail above**), the poking assay is also very sensitive to variations in poking probe size, positioning angle and indentation speed, which all have strong effects on inactivation time constants, as my lab has recently shown (Zeitzschel & Lechner, Channels 2024). Hence, the poking assay is far from being perfect and definitely not superior to the stretch assay with regards to measuring inactivation kinetics.

We agree, however, that the stretch-assay also has limitations – as stated by the reviewer – caused by seal creep, increased basal membrane tension and possibly tip geometry. In contrast to cell relaxation, which is the main confound in poking experiments that cannot be avoided, all afore-mentioned confounds of the stretch assay can and were minimized in our experiments. Thus, patch pipettes were pulled with the same parameter such that tip geometry and diameter only showed minimal variability across experiments. To minimize inter-experimental variability in basal membrane tension we first, established the all gigaseals using the same negative pressure of -10mmHg and applied a positive pressure of +5mmHg to minimize seal creep. Most importantly we used that high-speed pressure clamp from ALA Scientific, which is equipped with a feedback control that keeps the pressure at the setpoint

throughout the duration of the stimulus thereby minimizing adaptation. Therefore, we believe that after all the pressure-clamp assay is better suitable to assess PIEZO1 inactivation. An excellent review highlighting and critically discussing the pros and cons of the two techniques has recently been published by the Grandl lab (PMID: 34120715) and is cited in our manuscript.

We would also like to point out that the reviewer contradicts him/herself when claiming that *"While stretching is useful for measuring the Hg50, it is far less precise when it comes to measuring the timing of inactivation",* due the afore-mentioned confounds. I am sure the reviewer is aware that membrane tension is the actual stimulus that activates PIEZO1 and according to Laplace's law T = P*R/2, where T is tension, P is the pressure and R is the radius of the patch. Accordingly, a given pressure causes different membrane tension depending on the size and geometry of the patch pipette, which determines the radius of the membrane patch.

Also, we are not aware of any paper that has shown that the pipette geometry alters inactivation kinetics of PIEZO1 in the stretch assay and unfortunately the reviewer did not provide a reference that would support his/her statement. Maybe he/she had the recent study by Wang et al. in mind (PMID: 38951553), but this study examined pipette geometry-dependent cytoskeletal redistribution in the membrane patch and did not provide any information about its impact on channel inactivation.

We hope our detailed explanation together with our new AFM data could convince the reviewer that the stretch assay might after all be the better choice for assessing inactivation and that this point is no longer an issue.

Fig. 1C vs D. Quantification of peak/sustained in D (~2) doesn't seem to match the exemplary currents in C, where the ratio is closer to 1.
The peak/sustained ratios shown in Fig. 1 and 2 are averages of the ratios from the last three pressure pulses of each recording (i.e -50, -55 and -60 mmHg) and do thus not perfectly match the ratios seen in the largest currents examples. We have added this explanation to the method section and hope this resolves this issue.

Fig. 2. The authors convincingly show that the mutants slow inactivation. However, the conclusion that "in the flat (open) state – the wedge binds to the anchor-OH-linker…, thereby promoting rapid inactivation" is speculative (and so is the title of Fig 2). The authors refer to published structures, but there is nothing in the presented data that supports such a nuanced mechanistic conclusion. It would also be critical to test the importance of these mutants in the more sensitive poking assay.
In principle I agree that absence of evidence is not evidence of absence – in other words, just because the wedge has not been resolved in the curved state it doesn't mean that it is not there. However, the fact that three independent laboratories failed to resolve the wedge in the curved state, which indicates that it has a high mobility and cannot be captured by cryo-EM, whereas it was resolved bound to the anchor-OH-linker in the flat state resolved by the Xiao lab (PMID: 35388220), strongly supports the idea of state-dependent binding. This hypothesis is further supported by a recent preprint from the lab of Roderick MacKinnon, who is undoubtedly a very credible source for protein structures, which describes the flat PIEZO1 structure in cell-derived membranes and resolved the wedge within a few angström of the anchor-OH-linker in the flat conformation (Vaisey G. & MacKinnon R, bioRxiv 2025). The

relevant part of the PIEZO1 structure that binds the wedge is not shown in the preprint, but Prof. MacKinnon was so kind to share the PDB file of the structure with us even though it has not yet been released on the RCSB protein data bank, which also resolved the wedge in the same position just a few angström away from the anchor-OH-linker. Since Nat Commun publishes the review files alongside the manuscript, we cannot show the structure here for copyright and confidentiality reasons, because Prof. MacKinnons preprint has not been published and the structure has not been released yet, but to clarify this issue we have provided a separate image for the reviewer's eyes only.

My understanding of science is that we test hypothesis that emerge from previous work of others but that does not mean one has to repeat every single experiment on refers to. Hence, I don't quite understand why the reviewer has issues with the fact that we refer to published structures, especially considering that it is not a single structure we refer to but in fact four structures resolved by three independent laboratories.

Regarding the last sentence of this point i.e. "*It would also be critical to test the importance of these mutants in the more sensitive poking assay*.". For reasons explained in detail in our response to a previous point of this reviewer, the poking assay is clearly not more sensitive or suitable to measure inactivation. Since we nevertheless very much appreciate the reviewers input and expertise we have tested '*…these mutants*' (the reviewer doesn't specify which but we assume he/she means the P1.Y1442_W1445A and P1.Y2175A_K2184A mutants) in the poking assay and also observed a two-fold increase in the inactivation time constant (new Fig. S3), consistent with the effects of full and partial wedge deletion.

We hope this explanation together with the new data from additional poking experiment satisfy the reviewer and resolve this issue.

Fig. 2E. What the authors have really shown is that the wedge domain contains residues that influence the sensitivity of the channel to membrane stretch (decrease Hg50). This, rather than inactivation, could be its primary role.
I have to admit that I am a bit confused by this comment. In the previous point, the reviewer states that "*the authors CONVINCINGLY show that the mutants slow inactivation*" and here he/she says that rather than inactivation, the primary role of the wedge may be to change the sensitivity of PIEZO1. The reviewer contradicts him/herself and I am not quite sure what to reply to this comment. Yes, there is also an effect of Hg50 in the point mutants shown in Fig. 2, but why does the reviewer think that this could be the primary role of the wedge.
The reviewer completely ignores Fig. 3 where we show that PIEZO1 instantly recovers from inactivation in the absence of the wedge such that all channels are in a 'willing' state in which they are available for activation at any point in time. Full-length PIEZO1, by contrast, enters a 'reluctant' state after activation for a period of several seconds during which it cannot be activated. Accordingly, the fraction of full-length PIEZO1 channels that can be activated at any point in time depends on prior activity (e.g. background activity from traction forces or prior mechanical stimulation during a patch clamp recording). Hence, the more likely explanation is that the shift in P50 is an indirect consequence of the instant recovery from inactivation, which increases the proportion of available channels, and not the other way round.
We hope our explanation makes sense and resolves this issue.

Fig. 4. The data do not support the conclusion that the increased interblade distance necessarily implies that a higher proportion of the channels is in the inactivated conformation

(as opposed to 'closed', deactivated, or some other), or that it suggests the "ball-and-chain mechanism". The authors do not present any data that the wedge acts as a ball that plugs the pore, this mechanism is not tested and the conclusion is speculative. The decreased proportion of 'flat' channels is also consistent with the mild overall effect of the wedge on inactivation.

To address this point, we would like to first clarify the definition of **inactivation**, which is that channels enter a state in which they stop conducting ions and which renders them "refractory", meaning that they cannot be reopened by an normally activating stimulus until they recover from inactivation. Moreover, we would like to draw the reviewer's attention to Fig. 3 and fig. S4, which he/she seems to completely ignore in his/her evaluation of our manuscript and which clearly show that – in the absence of the wedge – PIEZO1 never enters such an inactivated state and remains fully activatable at any time. Thus, when fixing cells for MINFLUX imaging, it is highly unlikely that any PIEZO1-wedge^{DEL} channel would be captured in an inactive state, whereas numerous PIEZO1-WT channels would be, considering that recovery from inactivation takes several seconds. Accordingly, since the only conformational state that is more or less absent in the interblade distribution histogram of the P1.wg^{DEL} as compared to full-length PIEZO1 is the fully flattened conformation, we conclude that most of the fully flattened channels PIEZO1 are in an inactive state. The entire last section of the discussion of the original as well as the revised submission is dedicated to this point and we don't see how our explanation could be improved. Since the reviewer also didn't suggest any experiments that could clarify this issue or specify which data he/she would like to see to be convinced, we did not perform additional experiments to resolve this issue and stick to our original explanation.

Regarding the reviewers comment that we *"…do not present any data that the wedge acts as a ball that plugs the pore, this mechanism is not tested and the conclusion is speculative*", we would like to reemphasize our previous reply (see above). Thus, unlike stated by the reviewer, we neither claimed nor insinuated that the wedge *'…plugs the pore'* as stated by the reviewer. In fact, we had been very careful with our wording. We made it very clear that we think that the wedge serves as an inactivation particle that *"… terminates ion flux during sustained mechanical stimulation by binding to the anchor-OH-linker"* (line 292) or *"… contributes to inactivation by binding to the anchor-OH-linker in a ball-and-chain LIKE manner"* (line 333). Moreover, we would like to once again clarify that the ball-and-chain inactivation model of voltage-gated channels does NOT imply direct pore occlusion by the 'ball'. In fact recent research suggests that in voltage-gated sodium channels the 'ball' may bind adjacent to the pore and thus a novel and refined lock-and-key model has been proposed, as recently reviewed in detail by Bezanilla and colleagues (Liu et al. Nat Commun 2025, PMID: 41298396). The previously published cryo-EM structure of the flat PIEZO1 conformation (PMID: 35388220) and the afore-mentioned structure recently resolved by Vaisey & MacKinnon, clearly show that the wedge binds adjacent to the pore.

Regarding the final sentence of this point – i.e. *"The decreased proportion of 'flat' channels is also consistent with the mild overall effect of the wedge on inactivation"*, we have to admit that we simply do not understand what the reviewer wants to say with this. How is the decreased proportion consistent with mild effect? What's the logic behind this statement? Why mild effect? Earlier the reviewer stated that *"…the wedge domain apparently* ***eliminates*** *inactivation*" and "*The authors convincingly show that the mutants slow inactivation…*" and here its just a mild effect all of a sudden. I am very sorry, but I really do not know what and how to reply to this statement.

Minor

Fig. S2B It is not immediately obvious how these images convey the idea that the channels are on the plasma membrane. Please explain/elaborate.

Our intention was to provide images showing membrane expression of the indicated PIEZO1 mutants. We agree that these images were not ideal and have thus replaces them with stainings of unpermeabilized cells that clearly demonstrate membrane expression of the channels.

Line 111. The text implies that all three mutants were characterized by single-channel analysis, but the figure shows only one. Please correct.

We apologize for the lack of clarity. The reviewer is right, in that we had only recorded single channel activity from the P1.wedgeDEL mutant but not the other two partial wedge deletion mutants. To resolve this issue we have added single channel data from the partial deletion to the revised version of the manuscript and hope that this resolves this issue.

Reviewer #4 (Remarks to the Author):

I co-reviewed this manuscript with one of the reviewers who provided the listed reports. This is part of the Nature Communications initiative to facilitate training in peer review and to provide appropriate recognition for Early Career Researchers who co-review manuscripts.

Reviewer #4 (Remarks on code availability):

Did not review code.

Review of Paul Selvin:

In this study, the authors examined the wedge domain in PIEZO function using site- directed mutagenesis and electrophysiology and MINFLUX. They primarily compared the PIEZO1 to a deletion mutant P1.wg. They synthesized their data by arguing that a “wedge” sub- domain of the PIEZO1 acts like a “ball”, in a ball-and-chain model, to inactivate the proteins

Overall, I found the article about PIEZO1 to be interesting and to (partially) answer an important question in terms of activation by the so-called “wedge”. MinFlux was fairly impressively done although a more thorough analysis, answering my questions would be helpful.

I will primarily focus on their MinFlux results, as that is what I am an expert in.

- They must have a figure as how the PIEZO1 putatively closes. The introduction is nice but it’s difficult to follow with referring to a diagram. Fig 1A, second panel might be good enough, although I tend to think that the second panel should be the first panel (since it’s the most general) and it is unclear where the OH and IH are in the second panel and it is also unclear where the “beam” and “cap” is (on the second panel). Figure 5 is very useful.

  We thank Prof. Selvin for his constructive input to improve the intelligibility of Fig.1, although I have to admit that I am not quite sure if I correctly understand his question – specifically his comment regarding the regarding the location of the IH, OH, beam and cap in the ‘second’ panel – I assume Prof. Selvin refers to the image showing the flat structure of PIEZO1. The IH and OH are in the same position as in the left panel and are labelled

accordingly. The beam and the cap are not visible in either of the close-up views, because one would have to zoom out in order to see them and then important information about the location of the wedge would be lost. To resolve this issue we have highlighted the beam and the cap domains in the small top and side views and we have added a panel to supplementary Fig.1 showing larger regions including the cap and beam of PIEZO1 curved and flat from additional perspectives. We hope this additional information resolves this issue.

- On the spectroscopy part, there does not appear to be any results on DNA-PAINT! Maybe I've missed it, but the only reference (in the main or supplementary parts) to DNA- PAINT is Figure 4A.
- see reply below
- How do the results of MinFlux compare to that of DNA-PAINT?
- see reply below
- With DNA-PAINT you can get quantitative results, knowing when there is 1 vs 2 vs 3 fluorophores bound. (This is called Quantitative PAINT.) This could clear up any confusion. (It does involve knowing a single acceptor ton and toff, and for the PIEZO construct as well. The authors may already have this data.)

The three points above are best answered in a single reply. I am afraid there has been a misunderstanding regarding our experimental approach. Reading the second question ("*How do the results of MinFlux compare to that of DNA-PAINT?*") it seems like Prof. Selvin is under the impression that MinFlux and DNA-PAINT were two different experiments. This was not the case. We combined DNA-PAINT and MINFLUX in a single experiment as originally described by Ostersehlt et al. (2022, Nat Methods, DOI: 10.1038/s41592-022-01577-1) and as previously implemented in my lab for examining PIEZO1 conformation in its native environment (Verkest et al, 2025 Sci Advances, Verkest et al. 2025 Nat Commun). Thus, we did not perform classical camera-based DNA-PAINT but instead took advantage of the transient binding of the DNA-PAINT imager probes, which have binding times of app. 200 ms (according to Massive Photonics), to produce temporally and spatially scarce blinking events, the localisation of which can be precisely determined with the iterative TCP scan of MINFLUX. This technique has several advantages over classical MINFLUX, where stochastic photo-induced blinking of fluorophores is used, the most important of which is that photodamage of neighbouring fluorophores (e.g. three labelled blades of a PIEZO1 channel) is not an issue because there is a virtually infinite pool of fluorophore molecules in the buffer surrounding the cells. Thus, the sequential binding of DNA-PAINT imager strands to the three blades of a PIEZO1 channel, ensures that only one blade is labelled at a time such that photodamage or bleaching cannot occur, which significantly increases the detection rate of (sequentially) triple-labelled PIEZO1 trimers.

Accordingly, Prof. Selvins question about the *"…quantitative results, knowing when there is 1 vs 2 vs 3 fluorophores bound*" is not relevant for our experiments, because MINFLUX automatically filters out these events on the fly using CFR filtering, which is integrated into the scan sequence. CFR (center frequency ratio) is the ratio of the photon emission frequency at the center position of the MINFLUX excitation pattern over the mean effective emission frequency over all outer positions. When a single fluorophore is precisely localised (i.e. the fluorophore is in the center), the emission at the center position is close to zero, because there is no excitation light due to the donut shape of the excitation laser, and the CFR value is therefore also close to zero. When two or more fluorophores are simultaneously bound within the illuminated area, then the triangulated center position that

is wrong and the CFR values in the subsequent iteration will be above the cutoff such that the iterative TCP scan is automatically terminated (see figure below for further details).

2D MINFLUX iterative detection sequence

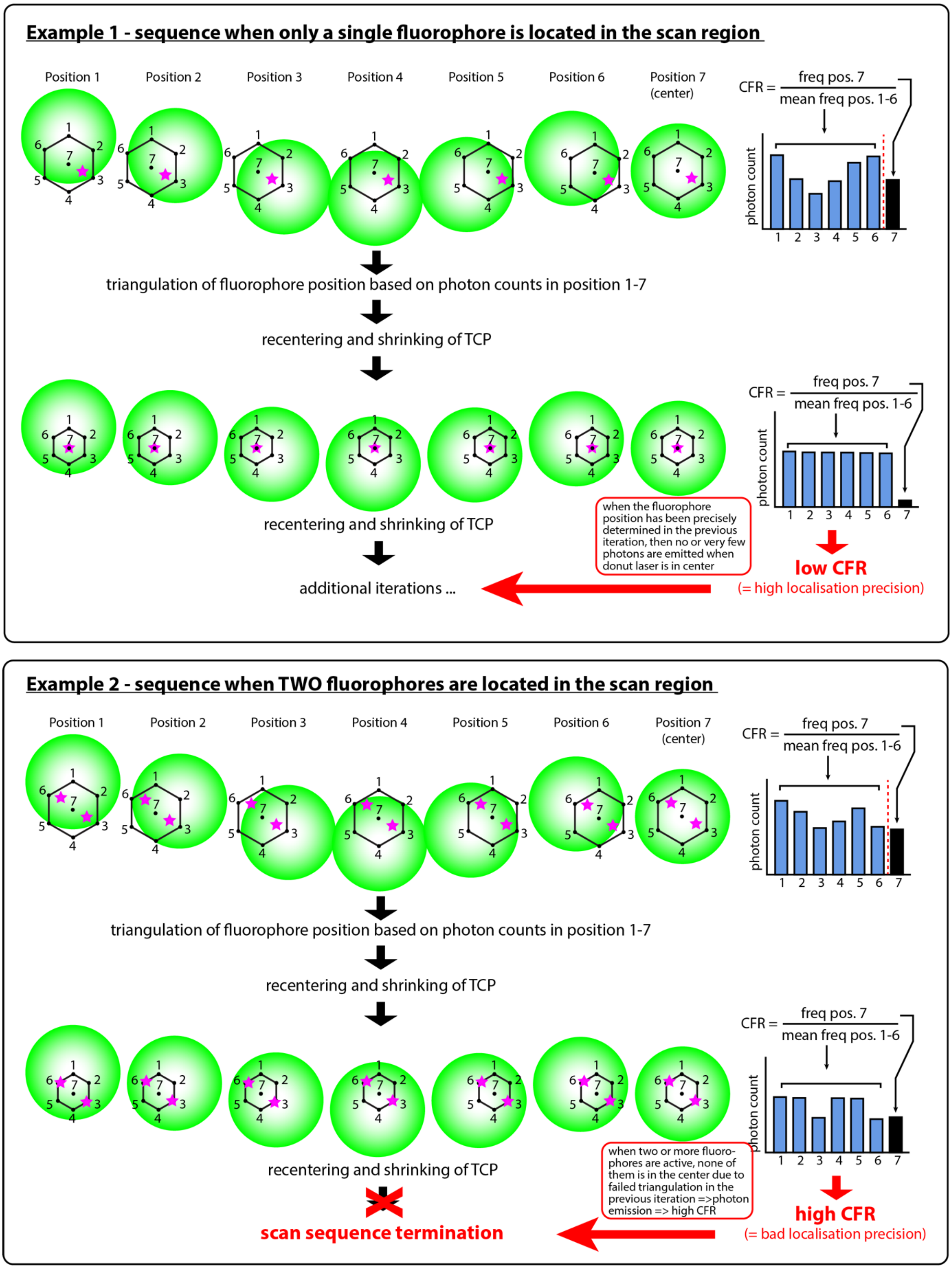

- In MinFlux, you get a difference of 3.59 nm. (Fig. 4C, right-side). What is the uncertainty in your data? I gather it's like 5 nm, and you must show that your statistics are good enough to make claims at this level.
  Assuming that all three localisations in a trimer (A, B and C) have the same isotropic localisation error of σ=4.16 nm (average of XYZ errors shown in fig. S5B), and applying the basic mathematical rules for error propagation, then the propagated uncertainty for the Euclidian distance between two points A and B is: $\sigma_d = \text{sqrt}(\sigma_A^2+\sigma_B^2)$ = 5.88 nm and the propagated uncertainty of the interblade distance, which is calculated as the average of the three distances (AB, AC and AB) in a triple labelled trimer is $\sigma_{interblade}$ = 5.88/sqrt(3) = 3.39 nm. Thus, the propagated uncertainty is slightly smaller than the difference of the means, which is good, but we agree that it may not be statistically meaningful. In this context it is, however important to note that the distribution of interblade distances shown in the scatter plot in Fig. 4C does not only result from the Minflux error but mostly from the fact that PIEZO1 exists in various conformational states that have different interblade distances (please see our response to your question regarding 6 parameters below for additional details and see our previous publication by Verkest et al. Sci Adv 2025 where we analysed the distribution on a much larger dataset of more than 500 PIEZO1 trimers). Hence, a simple comparison of the mean interblade distances is not meaningful, which is why we performed the additional analysis of the interblade distribution histogram shown in Fig. 5D. We hope this explanation clarifies this issue.

  On the other hand, in Figure S4B where the authors show the Gaussian fits in x,y,z are very impressive. I assume when they say sz = 3.35 nm, that what is meant is the SEM of the data?
  I assume the reviewer refers to our use of the lowercase Greek letter sigma (σz) when he writes sz. The answer is no; we use sigma (σ) to designate standard deviation, as commonly used in statistics, and not SEM (standard error of the mean) and it refers to the standard deviation of individual MINFLUX 'traces' – i.e. multiple localizations of the same fluorophore. Standard deviations were calculated in Matlab as follows (also see code deposited alongside the manuscript):

```
% this code line creates a vector named 'id_tid' that contains all
  unique trace ID from one recording
[~, ~, id_tid] = unique(traces_FILT(:,4));
% this code creates a 3 x 1 array with xyz-standard deviations using
  the accumarray command in combination with id_tid (see previous
  line) and @std (command for calculating standard deviation)
StDevALL=[accumarray(id_tid,traces_FILT(:,1),[],@std)
  accumarray(id_tid,traces_FILT(:,2),[],@std)
  accumarray(id_tid,traces_FILT(:,3),[],@std)];
```

- It seems rather strange (in Figure S4B, but elsewhere as well) that sz is < sx or y. In our experience with MinFlux, the z-dimension is worse than in x or y.
  This is an excellent point and I am happy to clarify it. My lab is in permanent exchange with application specialists from Abberior, which builds the MINFLUX microscope and is located in Goettingen not far away from Hamburg. They frequently visit our Imaging Core Facility to calibrate the system and implement new features and they pointed us (a few years back when we started with Minflux) to an important consideration regarding 3D-Minflux that is that it is important to take into account the effect of the refractive index mismatch on Z-coordinate positioning. Thus, when using oil-immersion objectives with #1.5 glass

coverslips and aqueous buffers, a correction factor of 0.7 must be applied to all raw recorded z-positions. This is not automatically done by the Imspector software or by Paraview or when exporting raw data and thus has to be done during data post-processing. The correction factor has been calculated using DNA nanorulers and confirmed with simulations by Gwosch et al. (2020, Nat Methods) and is also explicitly mentioned on the MINFLUX Wiki page hosted by Aberrior. Since the Minflux Wiki is only accessible for registered users and the calculation in Gwosch et al. is somewhat buried in the supplementary material, it can, however, easily be overlooked. Indeed, I have stumbled across several Minflux papers where the authors were not aware of this and did not take this important factor into account. It is not my intention to insinuate anything and as mentioned above I am aware that instructions regarding the necessity of Z-correction can easily be overlooked but given that the Z error is worse in your – Prof. Selvin – experience, I was wondering if the lack of Z-correction could be the reason for this.

- In Figure 4D, you have your 6-parameter fit: (6-parameters is an awful lot: are any of thre 6-parameters linked so that you really only have, for example, 3 free parameters?
  I agree that 6 parameters is an awful lot, but it is important to evaluate and analyse the distribution of interblade distances in the context of what is known about the structural heterogeneity of PIEZO1. PIEZO1 is very large and, more importantly, structurally remarkably flexible. One can think of PIEZO1 as a three-armed octopus that can independently move its three arms along the Z-axis and – importantly – also along XY. Thus, in addition to transitioning between a flat and a curved conformation, the peripheral blade domains also exhibit significant rotational flexibility, which had originally been proposed by Mulhall et al. (Nature 2023) based on super-resolution microscopy data, and had later been supported by elegant in-silico simulations from the Kalli and Beech labs (Smith et al. Sci Advances 2025), who introduced the concept of 'handshaking' where neighboring blades interact with each other, which has recently been confirmed by cryo-EM of the C. elegans PEZO-1 channel. Hence, all 6 parameters are free. I hope this answers the reviewer's question and resolves this issue.

- In Fig 4D, the authors have the blue, pink and red that undergoes significant peak height changes. In the pink and red, there does not appear to be a change in distance (true? You could put in the actual distances), while the blue (closed conformation) is "significantly" different. What does this say either about the fitting or the structures.
  You are right, there is no change in the distance in the pink and red but the peak height significantly changes indicating that the proportions of channels with large interblade distances is much smaller in P1.wgDEL than in full-length PIEZO1. You are also right in that the fits of the closed conformations are dirtier and positions of the individual blue peaks are shifted around. As explained in our reply to your previous comment, the different closed conformations result from single, double or triple 'handshaking' between neighboring blades, but despite the remarkable resolution of Minflux the differences in interblade distances between these closed sub-states are difficult to resolve.
  The key point of this data set is that is relevant for the take-home message of this study is, however, that significantly less flat channels (most likely 'inactivated' channels, pink and red population) are detected in P1.wg^{DEL} and we believe and hope the reviewer does so too, that this claim is well supported by our data, despite the fact the fits at the lower end of the distribution, which scratches the resolution limit of MINFLUX, being a little dirty.

- In MinFlux, are the PIEZO structures fixed? If not, how is the diffusion accounted for, not to mention the stage drift. If not fixed, are the structures stable over the time scale necessary to do the experiment? (I would imagine that these are, in fact, discussed and I simply did not realize it.)

  Yes, our structures (cells) are fixed with paraformaldehyde and glutaraldehyde, as described in line 821 of the original submission (method section). Hence, diffusion is not an issue.

  Regarding stage drift, this is routinely monitored and corrected by the Minflux software, based on the position of immobile gold bead fiducial markers that are deposited next to the cells. This is a standard feature that had already been implemented in the first commercially available Minflux microscopes from Abberior. In the past, a bigger problem was drift of the beam itself, which becomes problematic with temperature fluctuations and long acquisition times. Our Minflux device is, however, equipped, with the recently developed beamline monitoring system, which compensates beam drift and thus improves stability and localisation precision. We have added this technical information to the method section of the revised manuscript.

- What are the numbers and percentages that actually had 3 sites labeled? Maybe put this in the Figure legends.

  The numbers that had 3 sites labelled are the N-numbers that are already provided in the figure legends (105 for PIEZO1 and 103 for P1.wedge^{DEL}). The percentages are impossible to quantify because we do not know how many of the other signals originate from double-labelled trimers as opposed to two nearby single-labelled trimers. Hence, any calculation or estimation of percentages would not be meaningful.

Point-by-point response to the reviewer questions

Reviewer #1 (Remarks to the Author):

"even in the complete absence of inactivation, poking-evoked currents will still substantially decay during sustained indentation simply because the force acting on the cell declines resulting in deactivation of a considerable proportion of channels. In this context it is also noteworthy that the auxiliary PIEZO1 subunits MDFIC and MDFIC2, which are mentioned further below by this reviewer, also impair deactivation (i.e. closure of the channel upon stimulus removal)"
The new data added is very nicely rationalised and well presented. I disagree on the extension of this to the effect of MDFI/C2 on deact as this effect is strongly voltage dependent (and by definition must be an inact effect and not deact). But the point is well taken and the AFM data very strongly supports the authors interpretation.

"Also, we could not find any mention of CDH2 (aka N-Cadherin) in Koster et al."
Figure 2 shows data for CDH2 and supports the idea that cadherins have little to no effect on PIEZO1 inactivation hence my suggestion to add – "putative binding partners".

Overall all of the changes are well described and well thought out. I strongly support publication.

We thank the reviewer for taking the time to re-review our manuscript and are happy to hear that he appreciates our efforts to clarify his concerns and supports publication of our study.

Reviewer #1 (Remarks on code availability):

N/A

Reviewer #2 (Remarks to the Author):

My main criticism of this manuscript is that deletion of the wedge domain has only a mild effect on inactivation in the poking assay, making it unlikely to be the critical determinant.
I agree with the authors that the poking assay has its limits and confounds, such as deactivation. Yet the strikingly mild effect observed in the poking assay, compared to the other mutations and conditions that the authors enumerated in the rebuttal, suggests that the critical determinant may lie elsewhere in the channel. The authors argue that the apparent discrepancy between the assays is because of 'deactivation' during poking. While this is possible, there is also a tangible possibility that the results seen in the stretch assay reflect patch mechanics inherent to the method itself. Greater agreement between the assays would provide more confidence that this domain is indeed critical.
We had already provided a very detailed and comprehensive response to this point in the first revision and do not have anything to add. We also don't understand why the reviewer now suggests that there is a "*tangible possibility that the results seen in the stretch assay reflect patch mechanics inherent to the method itself*.". How could patch mechanics inherent to the method itself selectively change the inactivation kinetics of our mutants? Wouldn't an inherent error or limitation affect all recordings alike?

I am happy that the authors have taken this into consideration and toned down their language in the abstract (abolishes -> diminishes), and I'd encourage them to make similar adjustments throughout the manuscript (e.g. the Figure 1 legend still uses "abolishes").
We have changed the title of Figure 1 to "Wedge deletion abolishes inactivation of pressure-evoked PIEZO1 currents" and hope this resolves this issue.

The addition of a thorough discussion addressing the discrepancy between the two assays is also commendable. For balance, I would suggest including a few sentences on the limitations and confounds of the stretch assay (e.g., seal creep, increased basal membrane tension, and tip geometry).
We are happy to hear that the reviewer likes the new section of the discussion. This new section only emphasizes the fact that stimulus adaptation may contribute to current decay during sustained mechanical indentation, which is supported by the new experimental AFM data. We did not add more sentences about possible general confounds of the stretch assay because these possible confound have already been discussed in great detail elsewhere (see eg an excellent recent review by Lewis and Grandl (2021) and are not directly relevant for subject of inactivation discussed here.

Overall, this manuscript is overly definitive in assigning the ball-and-chain-like role to the wedge domain and in claiming its critical role in inactivation.
Here is the final paragraph of our discussion: “Hence, we propose a refined inactivation model for PIEZO1 where the wedge, in addition to the cap and inactivation gate of the inner helix, **contributes** to inactivation by binding to the anchor-OH-linker in a ball-and-chain-like manner.”. We disagree that this statement is – as stated by the reviewer – “overly definitive”, but in fact very diplomatic and careful and clearly states that the cap and inactivation gate of the inner helix are important and that the wedge CONTRIBUTES to inactivation.

That said, the actual data are very solid, the topic is highly important, and the study would be of great interest to the community.
We thank the reviewer for appreciating that our data is very solid and for eventually supporting publication.

Reviewer #4 (Remarks to the Author):

“The article makes interesting contributions to the broader understanding of PIEZO1. The authors use electrophysiology, and MINFLUX nanoscopy measurements to understand inactivation regulation of PIEZO1.

Upon preliminary inspection, the experimental design of the manuscript appears robust. However, closer inspection reveals that certain elements in the experimental design and the narrative require closer scrutiny.

The reviewer would recommend the article for publication provided the following concerns are addressed, either through explanations in the manuscript, or through additional control experiments. The comments have been annotated by line number.

Line 852 – 854: A few concerns with this sentence, and broadly, the MINFLUX methods section.
It appears that the reviewer refers to the original submission and not to the revised version, because the revised version does not contain line numbers. Most of the 3D-Minflux/DNA-PAINT method section, including lines 852-854, was completely re-written in the revised version and now contains all the detailed information the reviewer asked for. We hope that clarifies things.

1. Have a table showing the acquisition parameters for each iteration. It is slowly becoming standard, and good practice in MINFLUX literature. Look up the kinesin tracking paper from Jonas Ries's group, or Stefan Hell's group, to see how such a table should be formatted. Even if it is the manufacturer's default sequence file, the information relevant to that must be included.

A table describing the acquisition parameters has already been included in the revised version of our paper as supplementary table 2. This table contains information about all relevant parameters, such as pattern geometry and size, photon limit, background limit, dwell time, pattern repeat factors, stickiness, CFR limits and laser power factors.

Admittedly, we mixed up the tables and provided the one for measurements at 560 nm (which is used for another study in my lab) and not the one for 642 nm which was used here.

The only difference is, however, the pattern diameter (L) which is calculated as L = patGeoFactor*360*(wavelength/642). We have corrected the table accordingly in the final version of the paper and hope this resolves the issue.

2. How confident are the authors of the study that they used the manufacturer's default sequence file, with no alterations? In the reviewer's experience, (and from anecdotes from other users of the MINFLUX), the default sequence file of the manufacturer can be very difficult in providing 1 nm – 5 nm localization precisions, unless significant sequence file edits are done. If requirement 1 above is fulfilled, the reviewer would have greater confidence in the robustness of the MINFLUX acquisition method. The Methods section needs to illustrate how it was done, and the credibility of the measurement must be further bolstered.

We are very confident that we used the manufacturer's current default sequence file "Imaging_3D_Nov2022.json", which is the currently approved revision (2 July 2024) available on the abberior wiki page (https://wiki.abberior.rocks). This is why we stated this in the method section.

We are sorry to hear that the reviewer has difficulties to achieve localisation precisions between 1 nm – 5 nm. The accuracy of the Minflux microscope, however, does not only depend on the parameters in the scan sequence setting file, but also very much on other factors, such as proper beam calibration and alignment, efficient feedback-controlled stabilization (fiducial markers) and so on. As we describe in detail in the method section, our samples were post-fixed with PFA/glutaraldehyde after labelling with the anti-ALFA nanobodies and the fiducial markers (gold nanoparticle) were further immobilized with PLL, which is essential for the feedback-controlled stabilization system to work properly. Moreover, we are using the novel beam line monitoring system, which further improves localisation precision.

Hence, we took several measures to ensure that our samples do not move and used an up-to-date system, which may explain why we obtain localisation errors below 5 nm.

We would also like to point out that we find it a bit inappropriate to refer to '*anecdotes from others'* when reviewing a paper, especially when there is published data that shows otherwise. Thus, there are numerous published studies that have used MINFLUX DNA-PAINT with the default scan sequence settings that obtained localisation errors similar to ours and below 5 nm (e.g. Ostersehlt et al. Nat Methods 2022, PMID: 36050490; Clowsley AH et al. Nat Commun 2025, PMID: 41423693; Macarron-Palacios et al. Sci Adv 2025; PMID: 40053592; Domart F et al. bioRxiv 2025).

Regarding the last sentence of the above statement where the reviewer writes that the "*Method section needs to illustrate how it was done…*", we are not quite sure what he/she means by "*it"*. The revised version of our method section has an extended 3D-Minflux/DNA-Paint section (changes to original version were highlighted in yellow), which contains very detailed information and all the points I mentioned above. Also, the table with the scan sequence parameters had been provided. We hope this explanation resolves this issue.

3. Was the localization of <5 nm achieved with stationary fluorophores? i.e.: were stationary controls done to distinguish between fluorophore movement and instrument noise? The reviewer would recommend this as a confirmatory control study. Just put down ATTO655 on a coverslip, with no cells, no movement of the fluorophores, and record 3D images of the fluorophores. Use the same methodology that was previously used to calculate localization precision. See if 1 nm can still be achieved.

I must admit that I am a bit puzzled by this comment. I assume by 'movement' the reviewer means thermal jitter or wiggling of our probes, right? There is no other movement because we are working with **fixed cells** that are labelled with an anti-ALFA nanobody, which was subsequently **post-fixed** (anti-ALFA contains a FX-cassette for better post-fixation) and finally, after addition of the gold nanoparticle fiducial markers, the whole thing was coated with **PLL for further immobilization**. All these steps were described in detail in the method section. Of course, even under these conditions, some components of the cell, including our fluorophores, might still exhibit some degree of thermal jitter, but that is something we can anyway not change because we cannot cool down our samples to cryo-EM-like temperatures of -200 °C, where thermal jitter would be eliminated.

The reviewer further suggests that we should "*just put down ATTO655 on a coverslip…*" to measure instrument noise. This is, however, not a trivial control experiment as insinuated by the reviewer, because ATTO655 cannot simply be '*put down'* on the coverslip. Immobilizing ATTO655 would require plasma cleaning of glass coverslips, followed by functionalization of the glass surface as well as the ATTO655 dye and subsequent deposition of the dye. However, even if we would manage to do this, how could we ever be sure that the deposited ATTO655 molecules would truly not move? Thermal 'wiggling' would probably still occur, right! Moreover, a recent preprint from the Soeller lab (Clowsley et al., bioRxiv 2026) addresses precisely this issue using DNA origami, demonstrating that a rigorous assessment of instrument noise would constitute an entirely separate study and is therefore beyond the scope of a revision.

Finally, we do not really understand why it is important to distinguish between localisation error originating from instrument noise and error originating from fluorophore movement in the first place? What would this tell us and how would it change the interpretation of our data and the take-home message? Every experimental system has an error that is the sum of multiple errors. Even the Minflux instrument noise, which the reviewer is interested in, could e.g. be further split into errors originating from wabbly fiducial markers resulting in poor stage stabilization, thermal fluctuations in laser power, poor calibration and alignment of the donut shaped laser beam and so forth.

Knowing the contribution of each error would in principal be nice to know, but it would definitely not change the interpretation of our data and the take-home message and there is nothing we could do to improve the error, because, we cannot build a better Minflux microscope.

Finally, we also do not understand the last sentence of this comment "*See if 1 nm can still be achieved*.". Why does the reviewer use the word '*still*' here and where does the value 1 nm come from. Our localisation error is ~4 nm along xyz and we never claimed an error of 1 nm, so why should we prove that an error of 1 nm can be achieved?

We hope our detailed explanation convinced the reviewer that measuring instrument noise is (i) very difficult and way beyond the scope of a second revision and (ii) anyway not an important control experiment that could change the interpretation of our data and the take-home message of our paper. Thus, we hope the reviewer – even though we didn't measure instrument noise – still recommends our manuscript for publication.

4. How was the localization precision calculated? Or was it reported directly from manufacturer-provided widgets such as ParaFLUX or FilterFLUX? The methodology and equations used, must be specified.”

As stated in the method section of the original manuscript, all analyses were performed using custom-written code in Matlab, which is available at Github (see code availability statement) and we did not use ParaFLUX or FilterFLUX. We would also like to point out that we had already provided the relevant code snippet, that contains the lines that calculate the localisation error, in our previous point-by-point response to the reviewers comments. In brief, the localisation precisions along xyz, as common practice in the field, are the standard deviations of the x, y and z coordinates, respectively, of all localisations of a given Minflux ‘trace’ (see Fig. 4A for explanation of what a trace is). We have added a detailed explanation of how the localisation precision was calculated together with the equation to the method section of the final manuscript for even more clarity and hope this resolves this issue.

Review of Paul Selvin:

In this study, the authors examined the wedge domain in PIEZO function using site-directed mutagenesis and electrophysiology and MINFLUX. They primarily compared the PIEZO1 to a deletion mutant P1.wg. They synthesized their data by arguing that a “wedge” sub-domain of the PIEZO1 acts like a “ball”, in a ball-and-chain model, to inactivate the proteins

Overall, I found the article about PIEZO1 to be interesting and to (partially) answer an important question in terms of activation by the so-called “wedge”. MinFlux was fairly impressively done although a more thorough analysis, answering my questions would be helpful.

I will primarily focus on their MinFlux results, as that is what I am an expert in.

- They must have a figure as how the PIEZO1 putatively closes. The introduction is nice but it’s difficult to follow with referring to a diagram. Fig 1A, second panel might be good enough, although I tend to think that the second panel should be the first panel (since it’s the most general) and it is unclear where the OH and IH are in the second panel and it is also unclear where the “beam” and “cap” is (on the second panel). Figure 5 is very useful.
- On the spectroscopy part, there does not appear to be any results on DNA-PAINT! Maybe I’ve missed it, but the only reference (in the main or supplementary parts) to DNA-PAINT is Figure 4A.
- How do the results of MinFlux compare to that of DNA-PAINT?
- With DNA-PAINT you can get quantitative results, knowing when there is 1 vs 2 vs 3 fluorophores bound. (This is called Quantitative PAINT.) This could clear up any confusion. (It does involve knowing a single acceptor ton and toff, and for the PIEZO construct as well. The authors may already have this data.)
- In MinFlux, you get a difference of 3.59 nm. (Fig. 4C, right-side). What is the uncertainty in your data? I gather it’s like 5 nm, and you must show that your statistics are good enough to make claims at this level. On the other hand, in Figure S4B where the authors show the Gaussian fits in x,y,z are very impressive. I assume when they say σ_z = 3.35 nm, that what is meant is the SEM of the data?
- It seems rather strange (in Figure S4B, but elsewhere as well) that σ_z is < $\sigma_{x \text{ or } y}$. In our experience with MinFlux, the z-dimension is worse than in x or y.
- In Figure 4D, you have your 6-parameter fit: (6-parameters is an awful lot: are any of thre 6-parameters linked so that you really only have, for example, 3 free parameters?
- In Fig 4D, the authors have the blue, pink and red that undergoes significant peak height changes. In the pink and red, there does not appear to be a change in distance (true? You could put in the actual distances), while the blue (closed conformation) is “significantly” different. What does this say either about the fitting or the structures.
- In MinFlux, are the PIEZO structures fixed? If not, how is the diffusion accounted for, not to mention the stage drift. If not fixed, are the structures stable over the time scale necessary to do the experiment? (I would imagine that these are, in fact, discussed and I simply did not realize it.)
- What are the numbers and percentages that actually had 3 sites labeled? Maybe put this in the Figure legends.
